# Supplementary material for: Deregulated Gab2 phosphorylation mediates aberrant AKT and STAT3 signaling upon PIK3R1 loss in ovarian cancer
Source: Nat Commun. 2019 Feb 12;10:716. doi: 10.1038/s41467-019-08574-7 (PMC6372715; doi:10.1038/s41467-019-08574-7)
Supplement: Supplementary file 1 — Supplementary Information [file 41467_2019_8574_MOESM1_ESM.pdf]

Supplementary information

Deregulated Gab2 phosphorylation mediates aberrant AKT and STAT3 signaling upon *PIK3R1* loss in ovarian cancer

Xinran Li, Victor CY Mak, Yuan Zhou, Chao Wang, Esther SY Wong, Rakesh Sharma, Yiling Lu, Annie NY Cheung, Gordon B Mills, Lydia WT Cheung

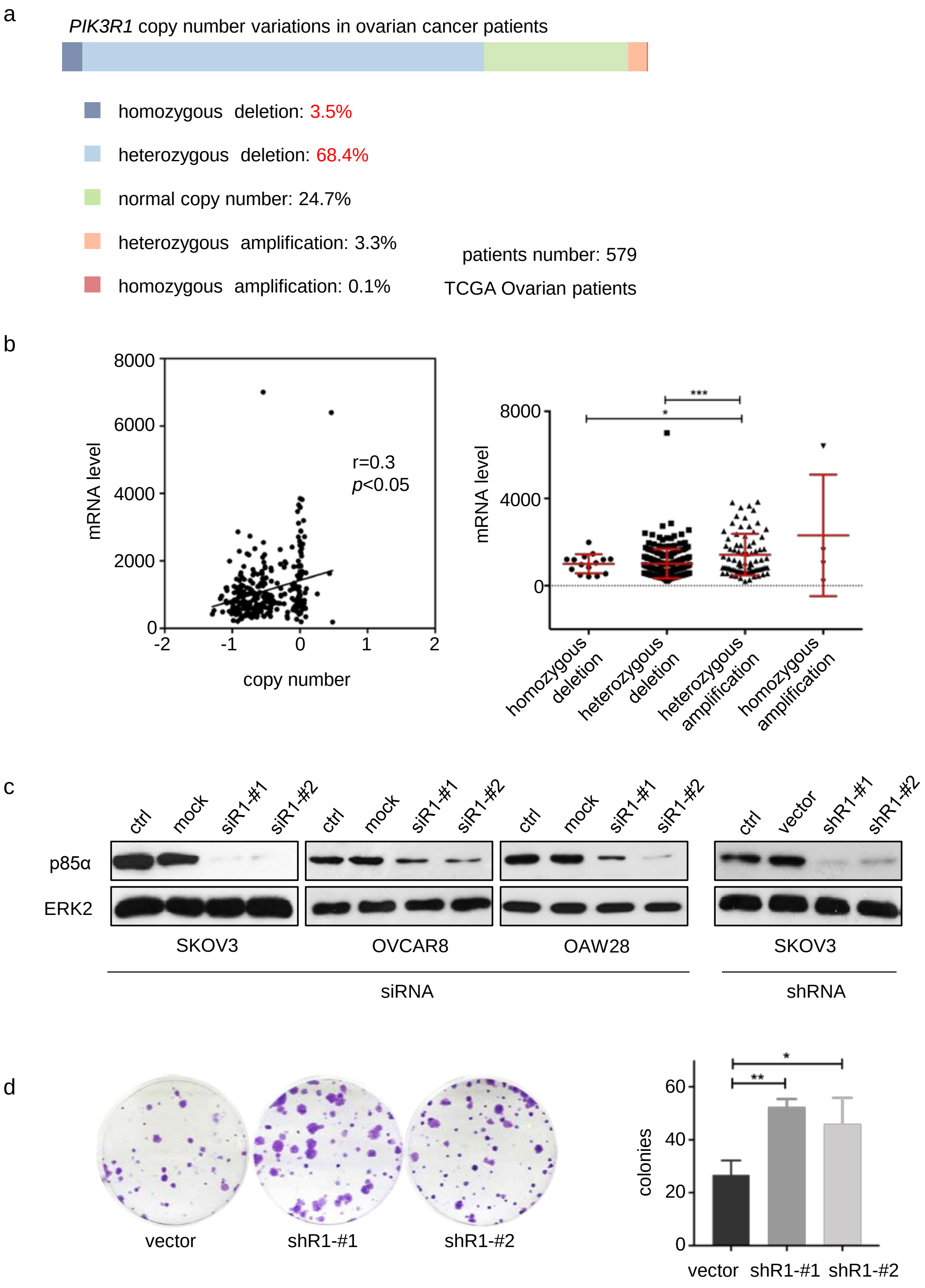

Supplementary Fig. 1. *PIK3R1* loss is frequent in ovarian cancer patients and is tumorigenic. a, The occurrence of *PIK3R1* copy number variations in TCGA ovarian cancer cohorts (n= 579). **b,** Correlation of *PIK3R1* copy number and mRNA levels in TCGA ovarian cancer patients. **c,** Knockdown efficiency of *PIK3R1* siRNA (left) and shRNA (right) was validated. **d,** Two hundred *PIK3R1* shRNA- or empty vector-stably expressing SKOV3 cells were seeded in 6-well plates and allowed to grow for 14 days. Cell colonies were stained with crystal violet. Mean values of colony numbers with SD are shown. *, $p<0.05$. **, $p<0.005$. ***, $p<0.001$ using t-test.

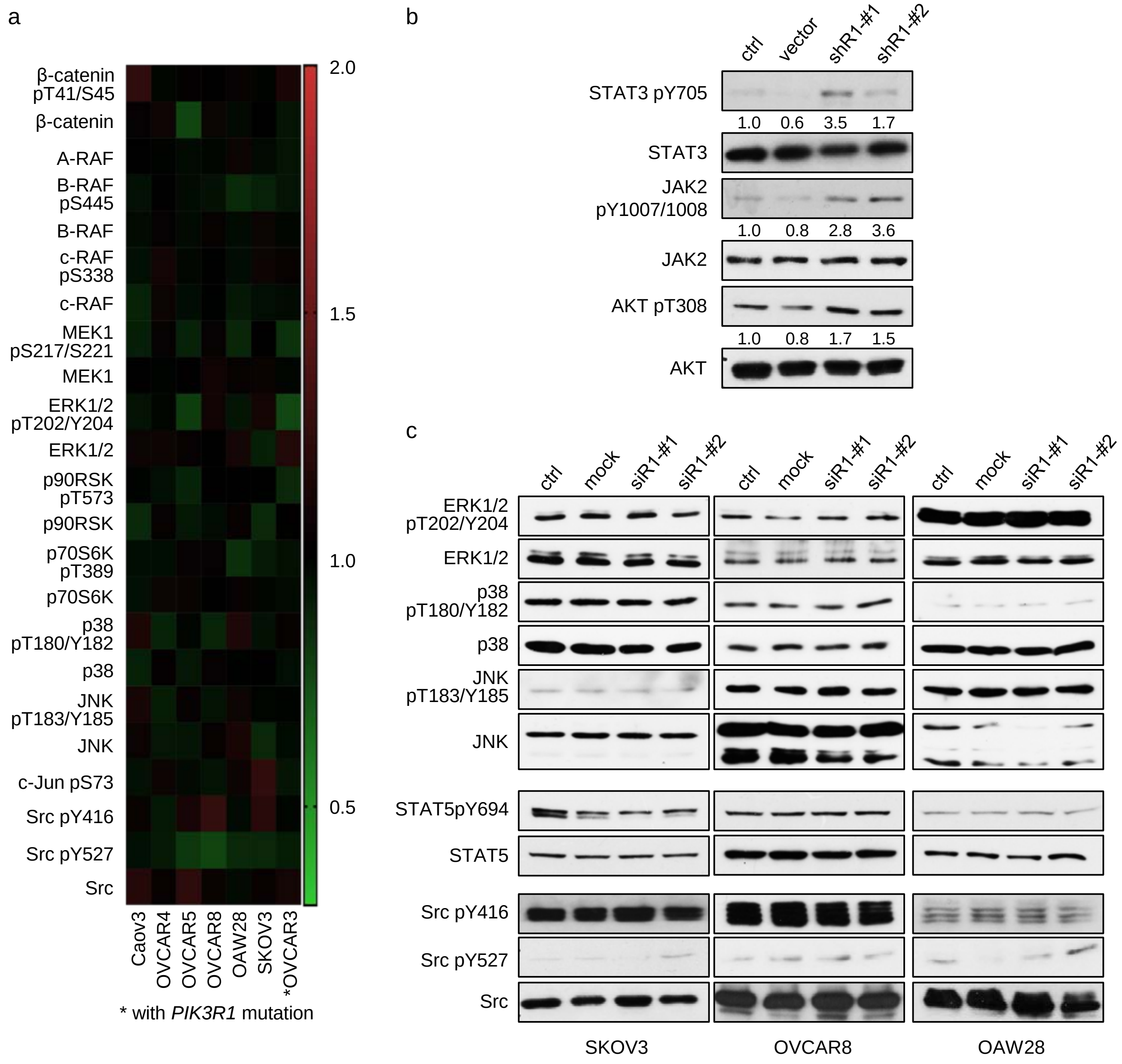
a
β-catenin pT41/S45
β-catenin
A-RAF
B-RAF pS445
B-RAF
c-RAF pS338
c-RAF
MEK1 pS217/S221
MEK1
ERK1/2 pT202/Y204
ERK1/2
p90RSK pT573
p90RSK
p70S6K pT389
p70S6K
p38 pT180/Y182
p38
JNK pT183/Y185
JNK
c-Jun pS73
Src pY416
Src pY527
Src
2.0
1.5
1.0
0.5
Caov3
OVCAR4
OVCAR5
OVCAR8
OAW28
SKOV3
*OVCAR3
* with PIK3R1 mutation
b
ctrl
vector
shR1-#1
shR1-#2
STAT3 pY705
1.0 0.6 3.5 1.7
STAT3
JAK2 pY1007/1008
1.0 0.8 2.8 3.6
JAK2
AKT pT308
1.0 0.8 1.7 1.5
AKT
c
ctrl
mock
siR1-#1
siR1-#2
ERK1/2 pT202/Y204
ERK1/2
p38 pT180/Y182
p38
JNK pT183/Y185
JNK
STAT5pY694
STAT5
Src pY416
Src pY527
Src
SKOV3
OVCAR8
OAW28

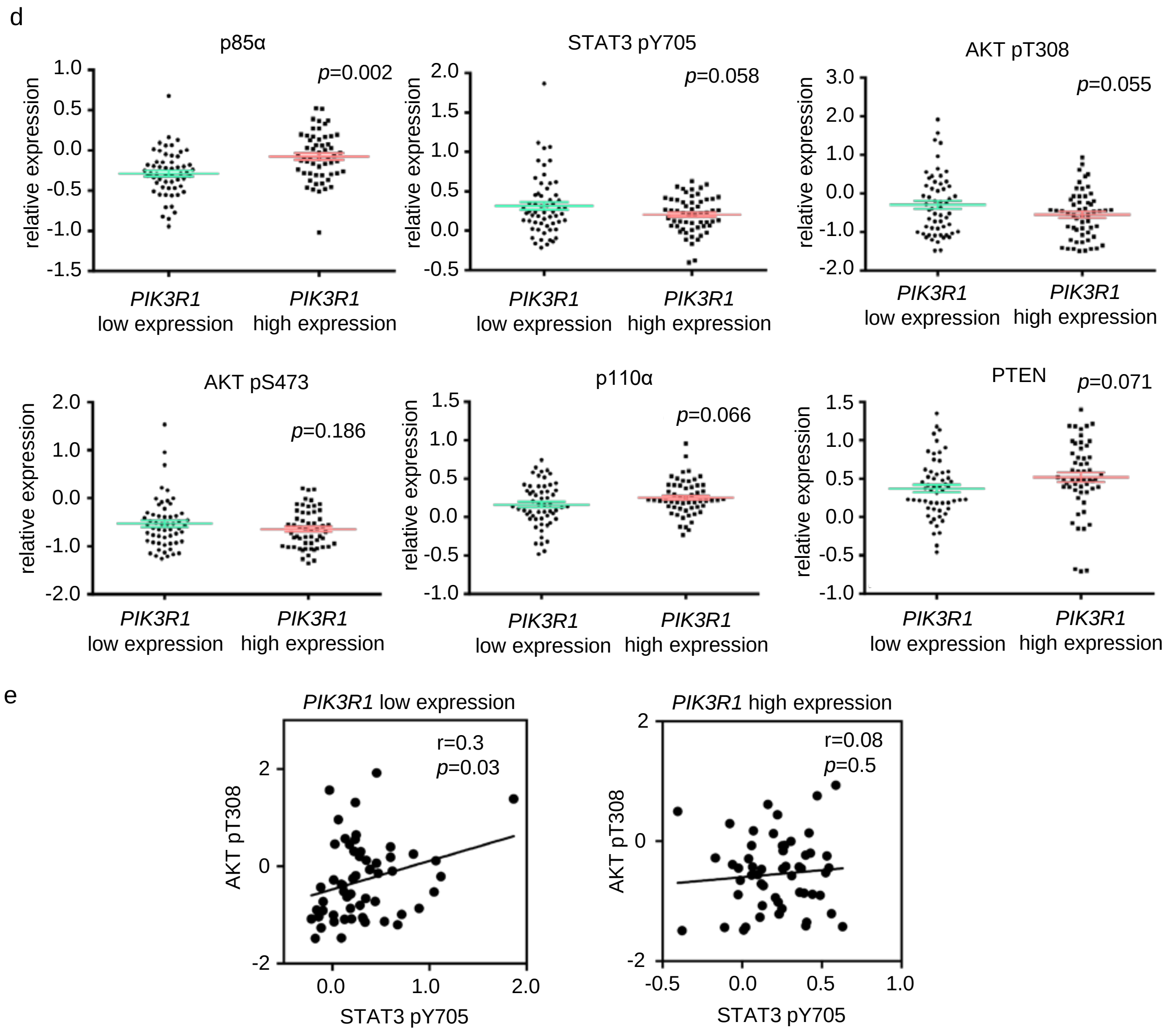

Supplementary Fig. 2. Activation of STAT3 and AKT in ovarian cancer cell lines or patients with *PIK3R1* loss. a, The levels of indicated proteins in 7 serous ovarian cancer cell lines transfected with *PIK3R1* siRNA (normalized to mock) determined by RPPA are shown as heatmap. **b,** Total lysates of *PIK3R1* shRNA- or empty vector-bearing SKOV3 cells or parental SKOV3 cells (control) were harvested for western blotting. The number below the band represents the mean value from densitometry readings of 3 independent experiments. **c**, Total lysates of transfected SKOV3, OVCAR8 or OAW28 were analyzed by western blotting. **d,** TCGA ovarian cancer samples were split based on *PIK3R1* mRNA levels. Levels of indicated proteins were compared between samples with low (first quartile) and high (4th quartile) *PIK3R1* mRNA expression. The *p* values were calculated by nonparametric Mann-Whitney tests. **e,** Pearson correlation coefficients between levels of phosphorylated STAT3 and phosphorylated AKT were calculated among *PIK3R1*-low (left) or *PIK3R1*-high (right) samples.

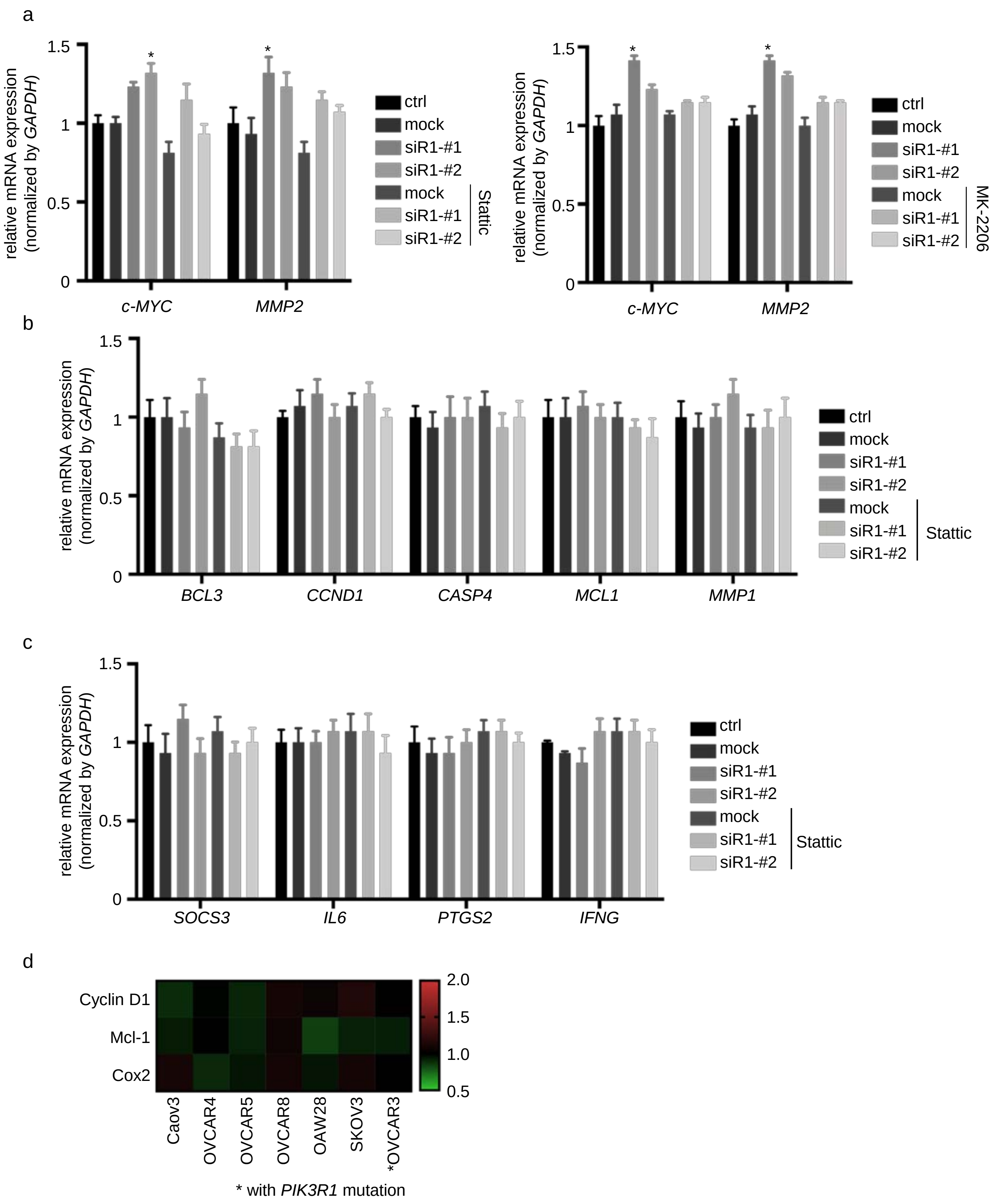

Supplementary Fig. 3. Real time PCR analysis of known STAT3 target genes in ovarian cancer cells with *PIK3R1* loss. a-c, Total RNA of siRNA-transfected SKOV3 treated with Stattic (10 μM) or MK-2206 (2 μM) or DMSO was collected for real-time PCR. *c-MYC* and *MMP2* levels are shown in **(a)**. Genes without significant change in mRNA levels are shown in **(b)**. mRNA levels of inflammation related genes are shown in **(c)**. **d,** Protein levels of Cyclin D1, Mcl-1, Cox2 determined by RPPA are presented as heatmap. *, p<0.05 compared with mock using t-test. Error bars represent SD.

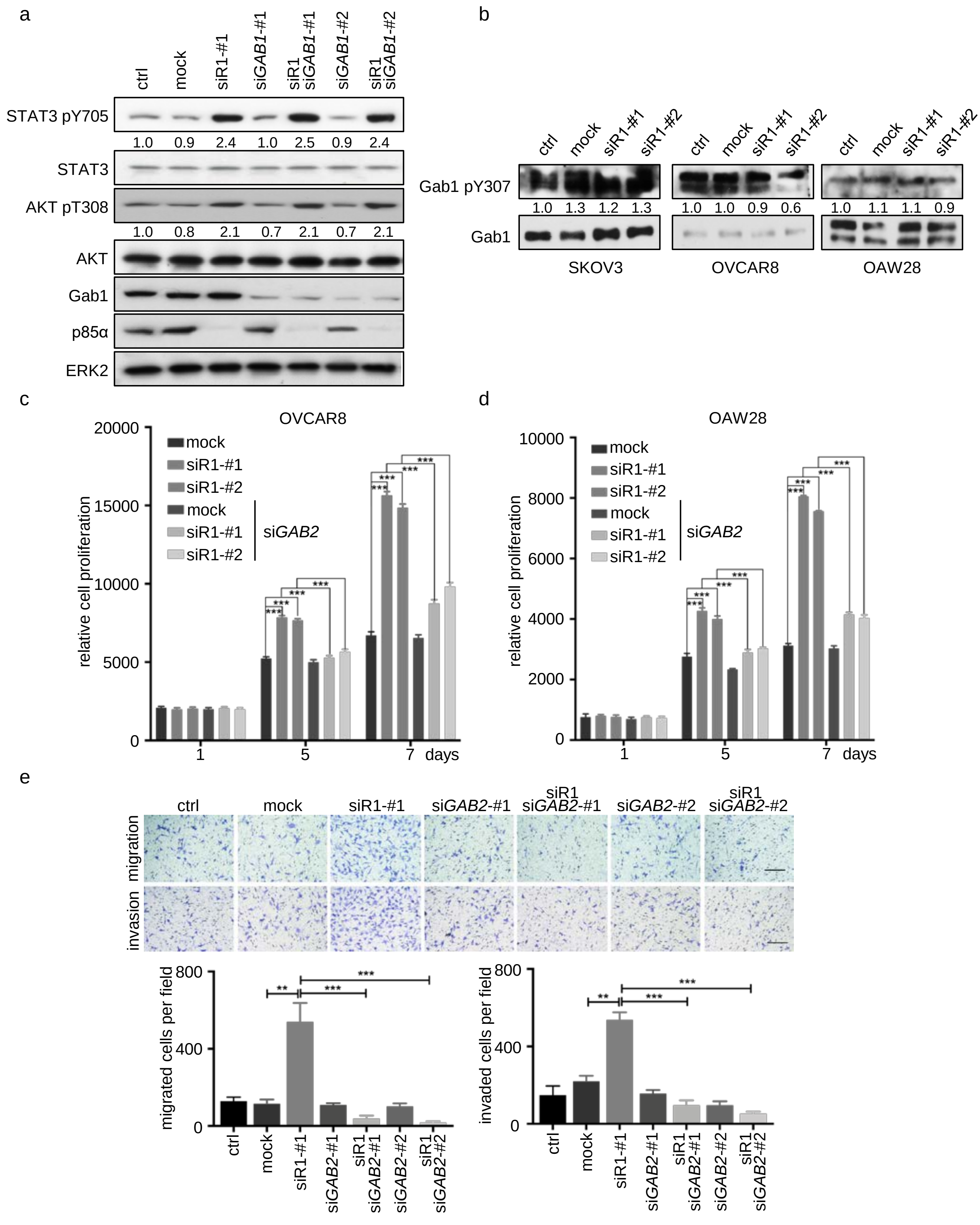

Supplementary Fig. 4. *GAB2* siRNA, but not *GAB1* siRNA, abolishes *PIK3R1* loss-induced tumorigenic properties. a, Total cell lysate of SKOV3 cells transfected with either *PIK3R1* siRNA, *GAB1* siRNA or in combination for 72 hr were harvested for western blotting. **b**, SKOV3, OVCAR8 and OAW28 cells were transfected with siRNA for 72 hr prior to western blotting. **c,** OVCAR8 or **d,** OAW28 cells were transfected with *PIK3R1* siRNA or co-transfected with *GAB2* siRNA, cell viability was measured over 7 days. **e,** Representative images of migrated or invaded OAW28 cells of 5 fields at magnification of 100x (upper) and means of migrated or invaded cells with SD of 5 fields at magnification of 100x (lower). Scale bar, 200 μm. The number below the band represents the mean value from densitometry readings of 3 independent experiments. **, *p*<0.005; ***, *p*<0.001; ns, no significant difference compared with mock using t-test. Error bars represent SD.

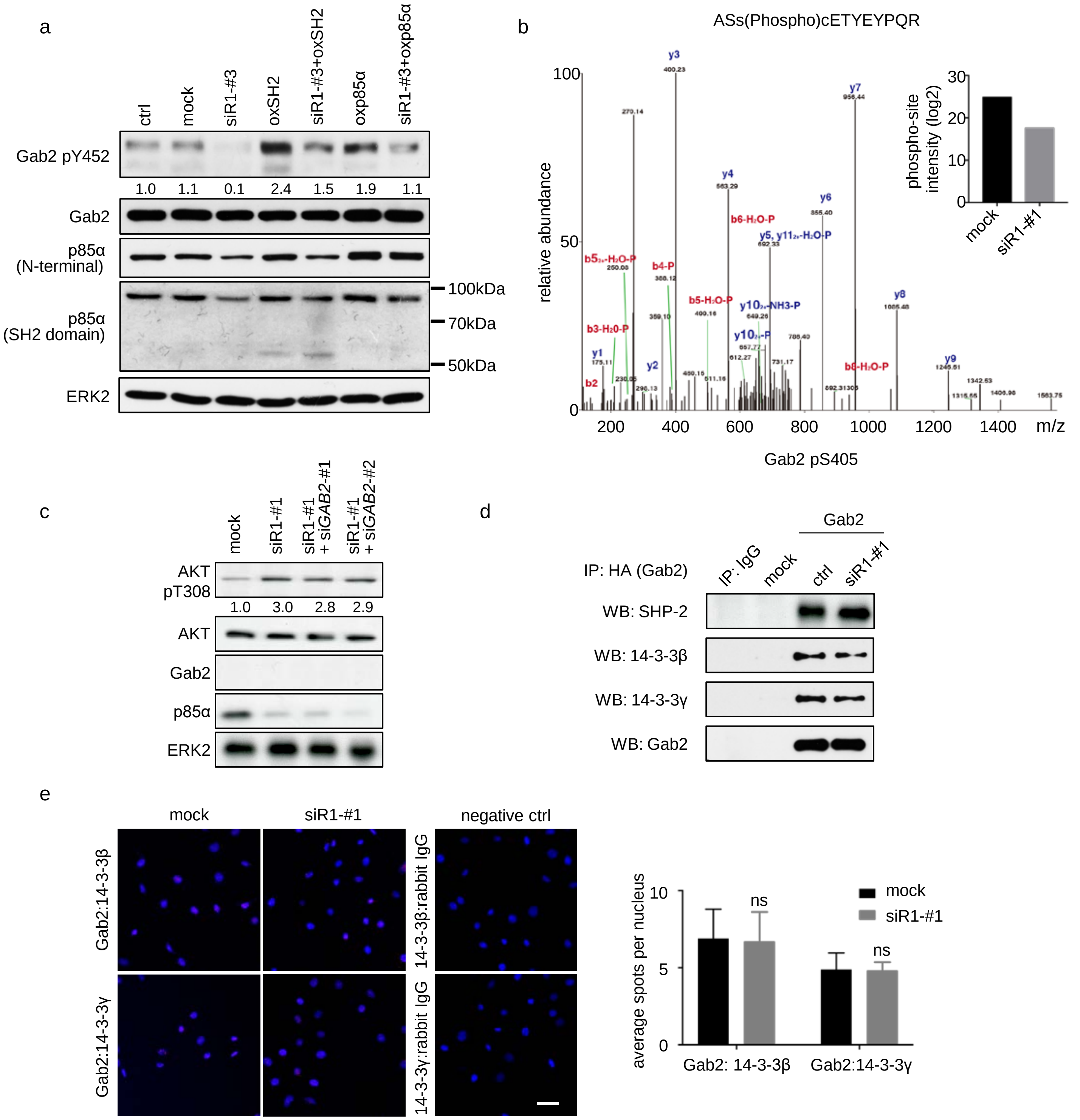

Supplementary Fig. 5. *PIK3R1* depletion leads to change in Gab2 phosphorylation but not in interaction of Gab2 with 14-3-3 or SHP-2. a, SKOV3 cells were transfected with either *PIK3R1* siRNA (siR1-#3, target 3'UTR of endogenous *PIK3R1*), overexpression (ox) with p85α SH2 domain, full-length p85α or in combination were subjected to western blotting. **b**, Mass spectrometry-based quantitative phosphoproteomics was employed to detect changes in Gab2 phosphorylation upon *PIK3R1* loss. The MS/MS spectrum of phosphopeptide containing the S405 phosphorylated residue of Gab2 is shown. Inset, the log2 intensities of S405 phosphorylation in the samples. **c,** OVCAR5 cells were transfected with *PIK3R1* siRNA or combined with two distinct *GAB2* siRNA prior to western blotting. **d,** Protein lysates of OVCAR5 cells co-transfected with HA-tagged *GAB2* overexpression plasmid and *PIK3R1* siRNA were immunoprecipitated with HA antibody and analyzed by western blotting. **e**, SKOV3 cells were transfected with *PIK3R1* siRNA prior to proximity ligation assay using the indicated pairs of antibodies. Representative images were presented with number of averaged spots per nucleus. Scale bar, 50 μm. The number below the band represents the mean value from densitometry readings of 3 independent experiments. ns, no significant difference compared with control using t-test. Error bars represent SD.

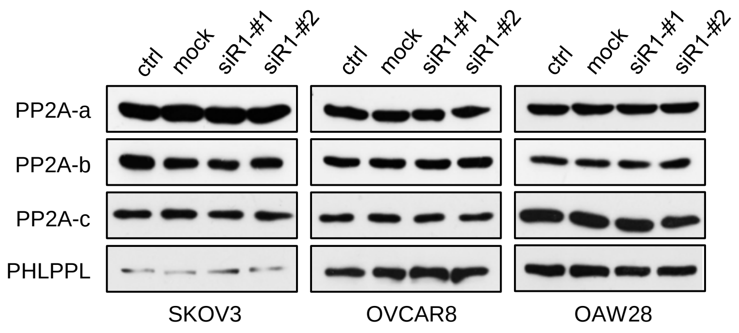

Supplementary Fig. 6. *PIK3R1* loss does not affect the protein levels of AKT-inactivating phosphatases. SKOV3, OVCAR8 and OAW28 cells were transfected with *PIK3R1* siRNA for 72 hr and protein lysates were subjected to western blotting.

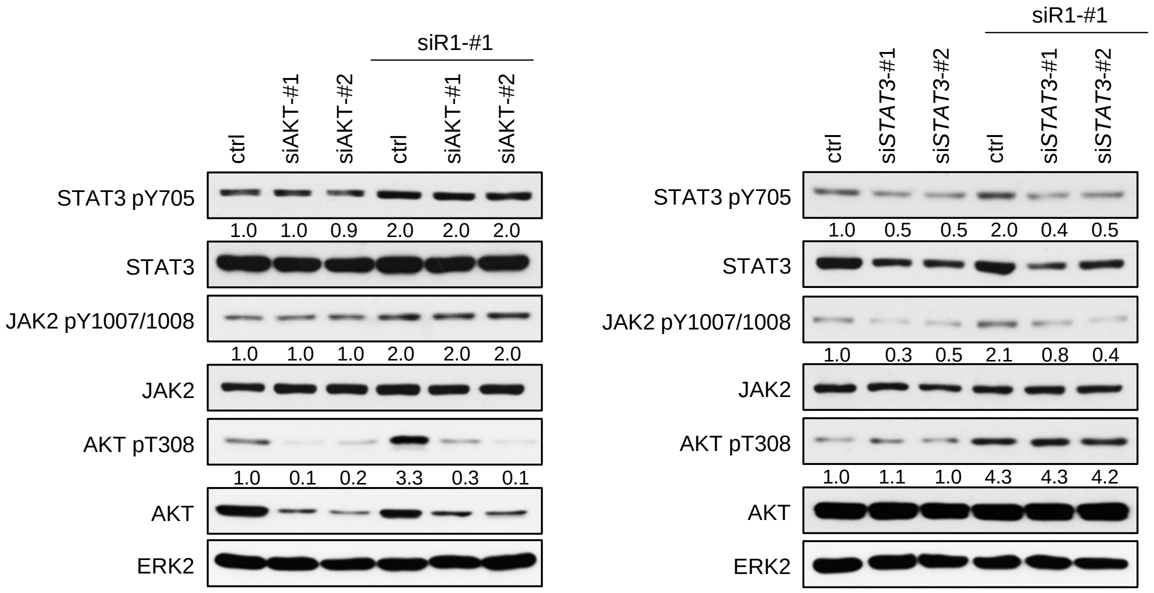

Supplementary Fig. 7. Activation of STAT3 and AKT signaling by *PIK3R1* loss is independent. SKOV3 cells were treated with *PIK3R1* siRNA 24 hr prior to *AKT1/2/3* (left) or *STAT3* (right) siRNA for 48 hr. Protein lysates were subjected to western blotting. The number below the band represents the mean value from densitometry readings of 3 independent experiments.

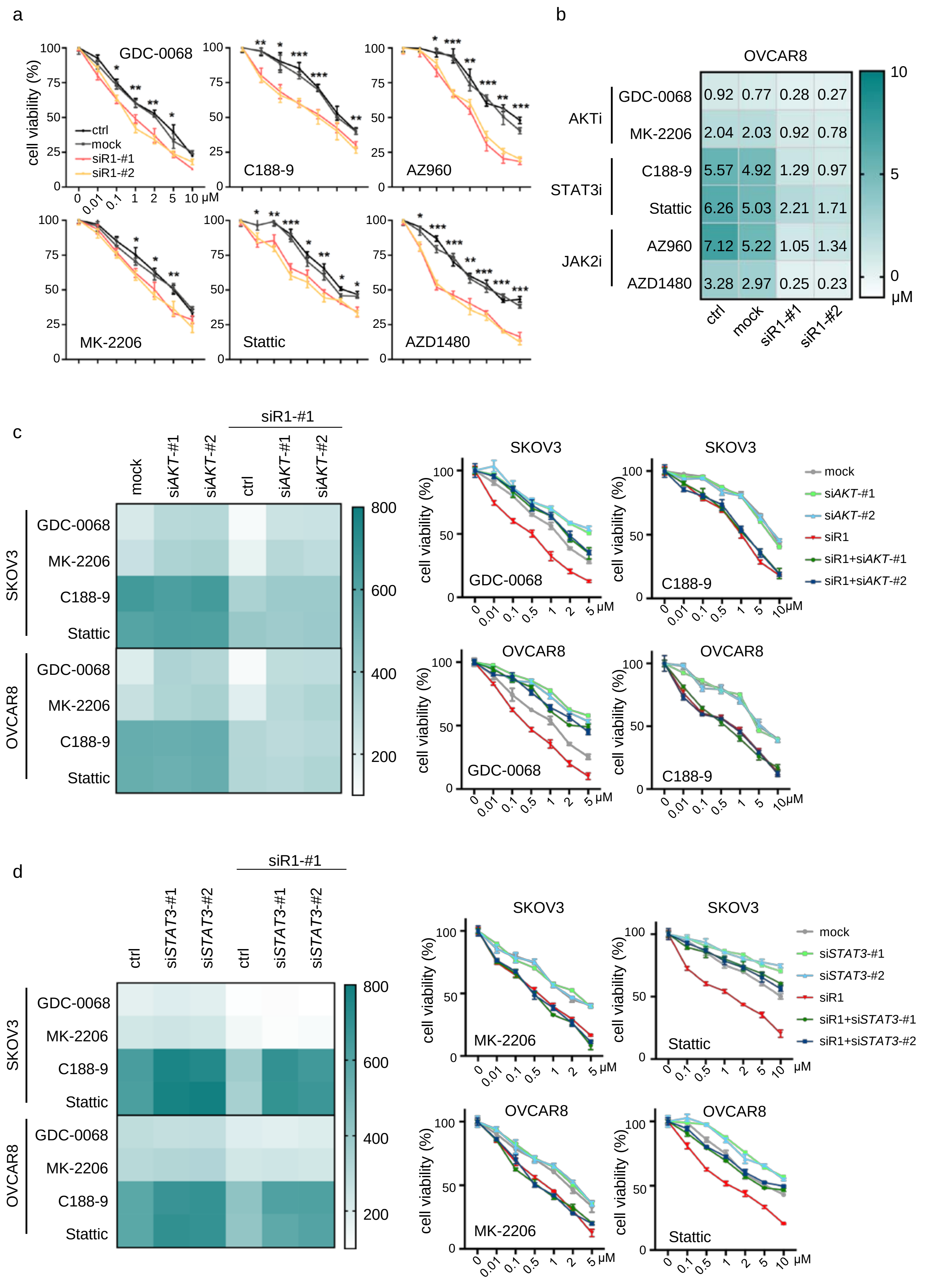
a
cell viability (%)
GDC-0068
C188-9
AZ960
MK-2206
Stattic
AZD1480
ctrl
mock
siR1-#1
siR1-#2
0
0.01
0.1
1
2
5
10 μM
b
OVCAR8
AKTi
STAT3i
JAK2i
GDC-0068 0.92 0.77 0.28 0.27
MK-2206 2.04 2.03 0.92 0.78
C188-9 5.57 4.92 1.29 0.97
Stattic 6.26 5.03 2.21 1.71
AZ960 7.12 5.22 1.05 1.34
AZD1480 3.28 2.97 0.25 0.23
ctrl
mock
siR1-#1
siR1-#2
10
5
0
μM
c
siR1-#1
mock
siAKT-#1
siAKT-#2
ctrl
siAKT-#1
siAKT-#2
SKOV3
OVCAR8
GDC-0068
MK-2206
C188-9
Stattic
800
600
400
200
SKOV3
OVCAR8
cell viability (%)
GDC-0068
C188-9
mock
siAKT-#1
siAKT-#2
siR1
siR1+siAKT-#1
siR1+siAKT-#2
d
siR1-#1
ctrl
siSTAT3-#1
siSTAT3-#2
ctrl
siSTAT3-#1
siSTAT3-#2
SKOV3
OVCAR8
GDC-0068
MK-2206
C188-9
Stattic
800
600
400
200
SKOV3
OVCAR8
cell viability (%)
MK-2206
Stattic
mock
siSTAT3-#1
siSTAT3-#2
siR1
siR1+siSTAT3-#1
siR1+siSTAT3-#2

e

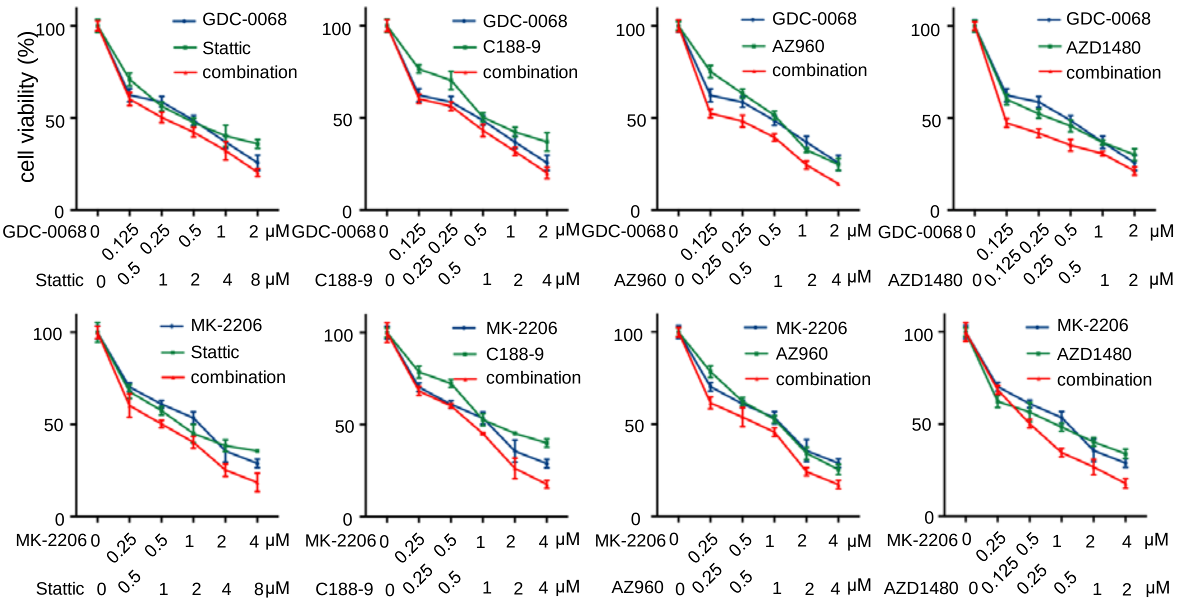

f

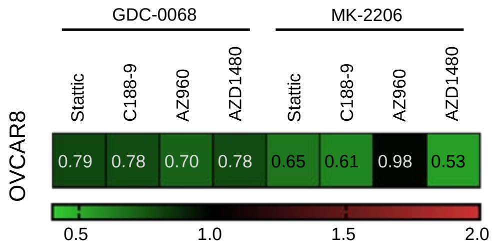

Supplementary Fig. 8. STAT3 and AKT inhibitors inhibit the viability of 3D spheiod of *PIK3R1*-depleted ovarian cancer cells and induce drug synergism. a, OVCAR8 transfected with *PIK3R1* siRNA were allowed to form 3D spheroids prior to treatment with indicated inhibitors for 72 hr. Dose response curves of each inhibitor are shown. **b,** IC50 values of each inhibitor were calculated by nonlinear regression analysis. **c-d**, SKOV3 and OVCAR8 transfected with indicated siRNA were treated with indicated inhibitors for 72 hr. AUC value of each dose response curve was calculated and is shown in the heatmap. **e,** OVCAR8 3D spheroids were treated with each inhibitor alone or in combination as indicated, dose response curves are shown. **f,** Combination Index (CI) values were calculated with Chou-Talalay Methods and CI values at the highest doses of the inhibitors are shown as heatmap. *, p<0.05; **, p<0.005; ***, p<0.001 compared with mock using t-test. Error bars represent SD.

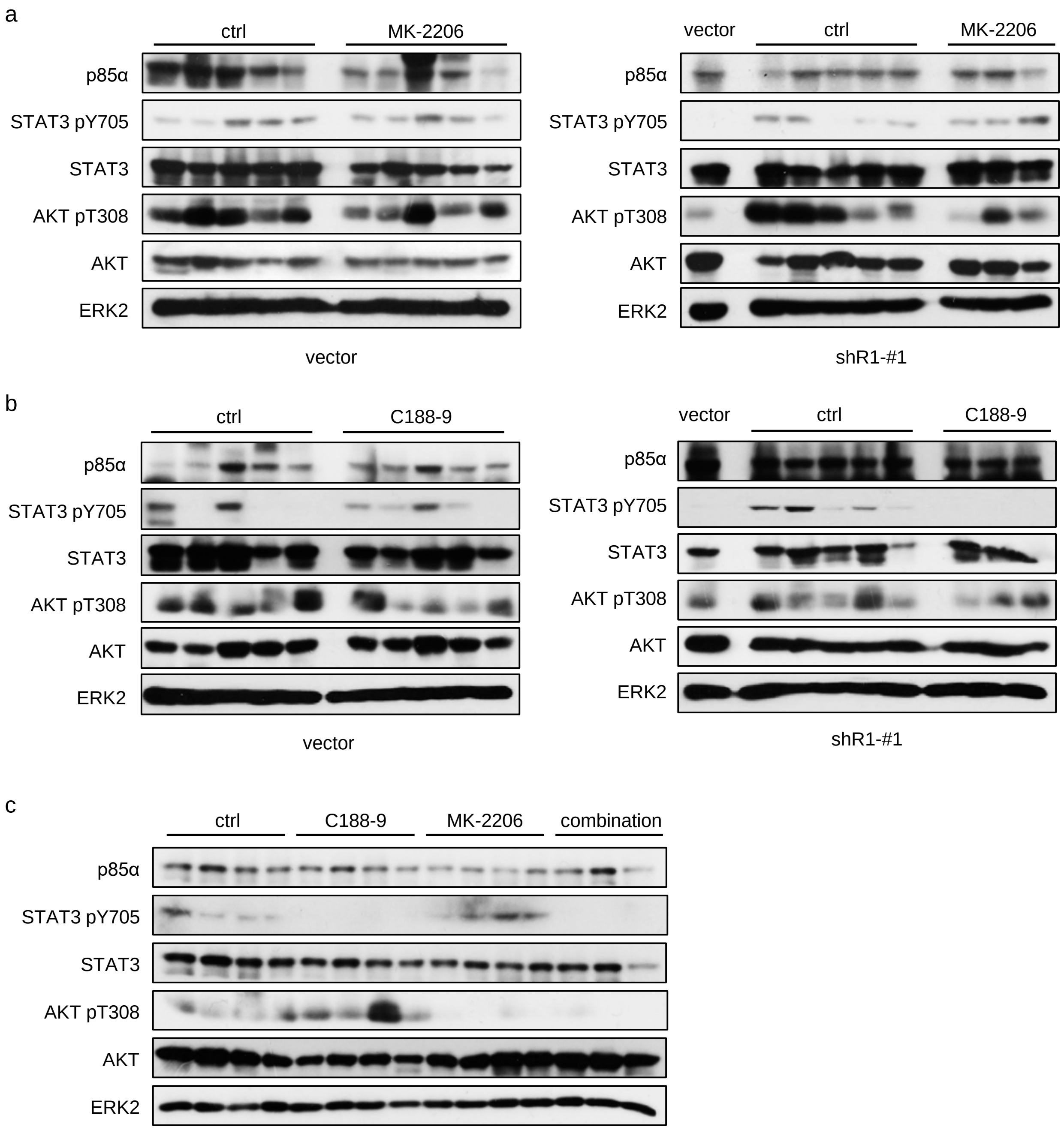

Supplementary Fig. 9. The STAT3 and AKT inhibitors effectively inhibit the corresponding pathways in ovarian tumor xenografts. a-c, Tumor nodules harvested were lysed with RIPA buffer. The inhibition of AKT by MK-2206 **(a)**, STAT3 by C188-9 **(b)** and combination of MK-2206 and C188-9 **(c)** *in vivo* were verified using western blotting. Tumor nodules were absent or were too tiny for protein extraction in some mice of the treated groups.

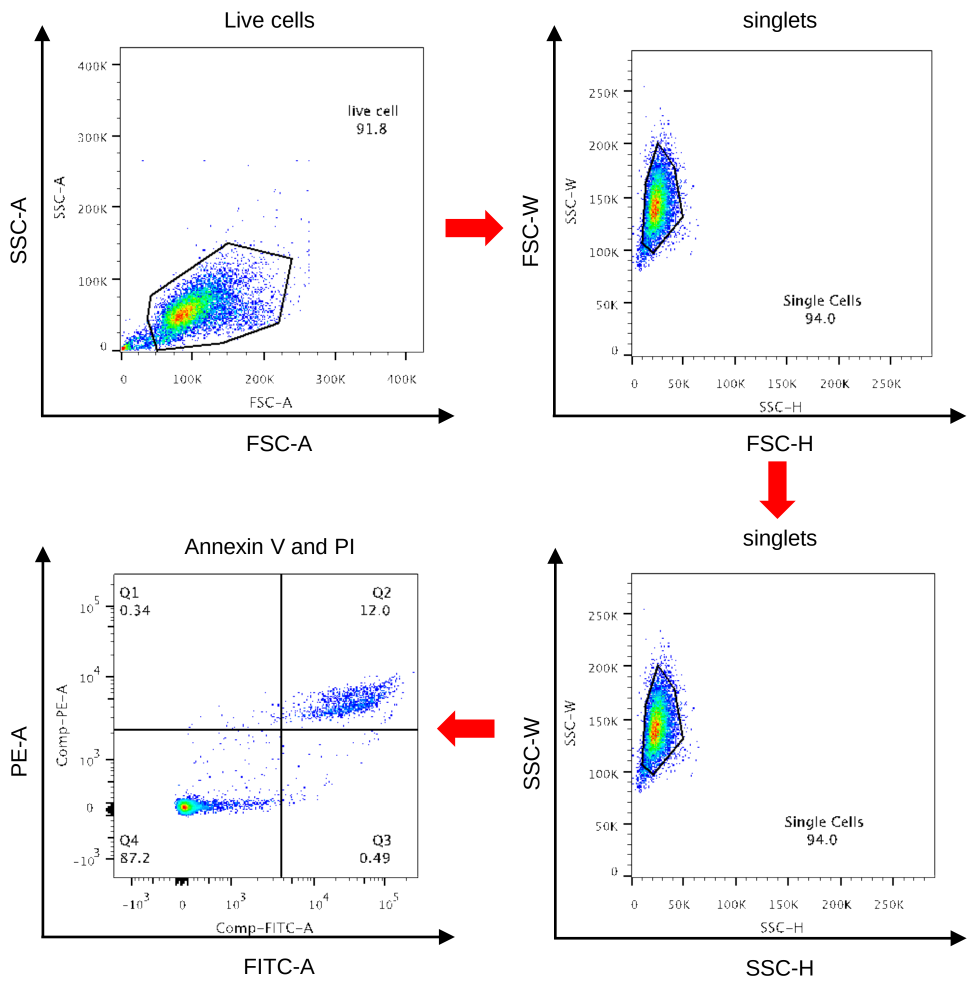

Supplementary Fig. 10. Gating strategy of flow cytometry for the data presented in Fig. 1c.

Figure 2b

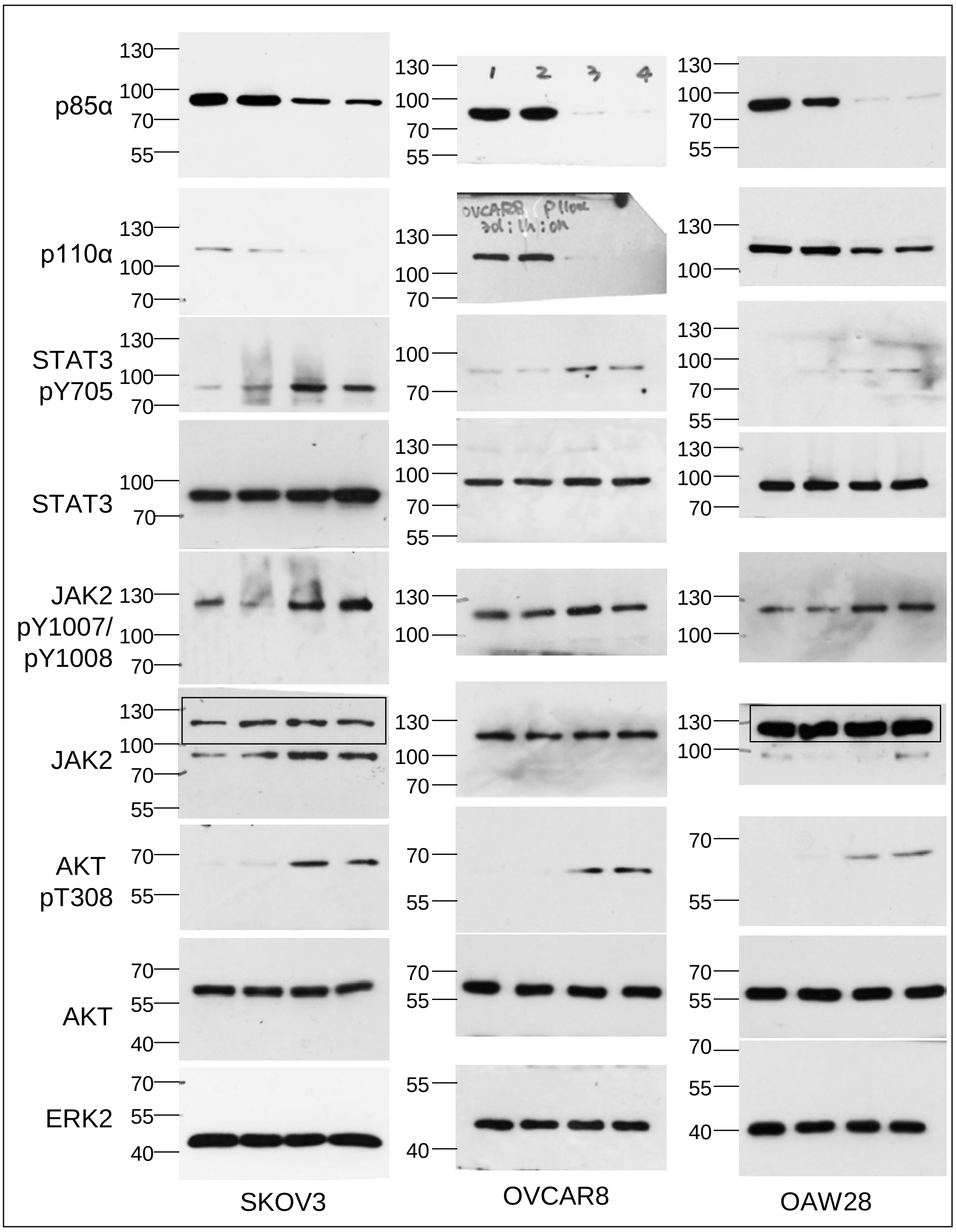

Figure 2c

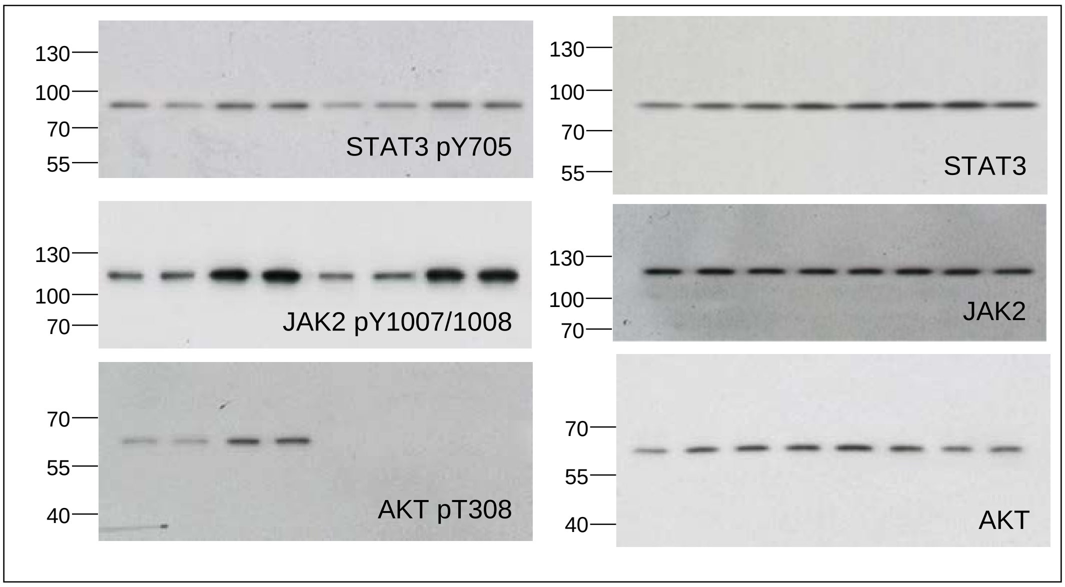

Figure 2e

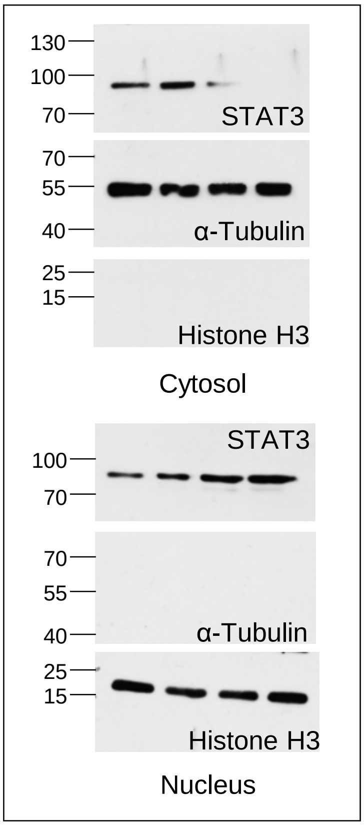

Supplementary Fig. 11. Uncropped western blots of Fig. 2.

Figure 3a

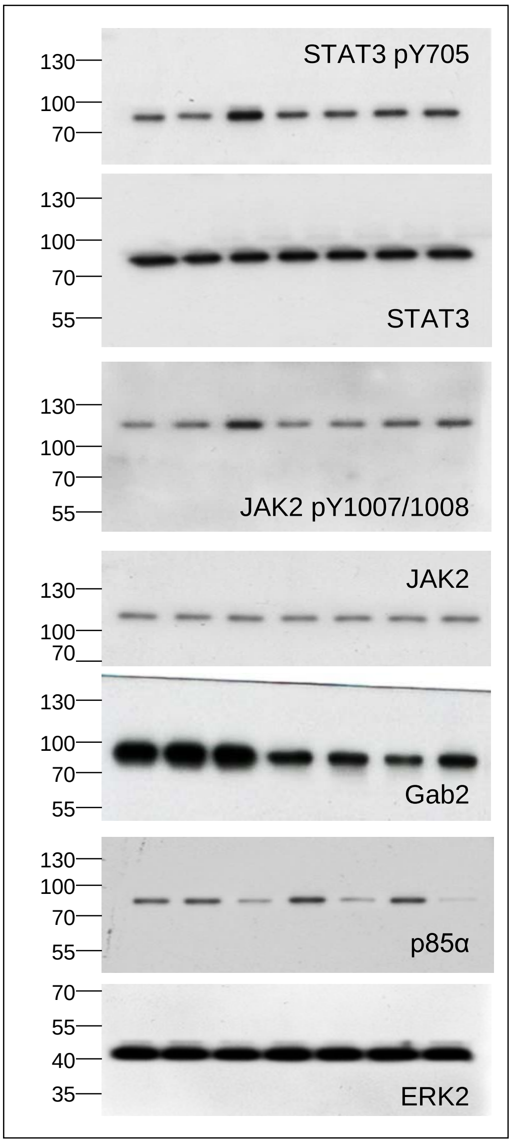

Figure 3f

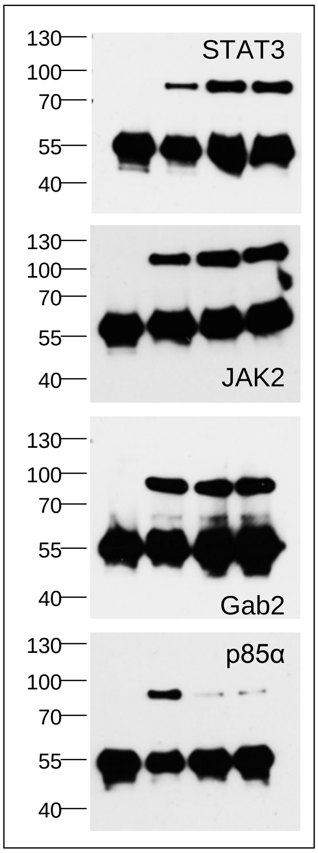

Supplementary Fig. 12. Uncropped western blots of Fig. 3.

Figure 4a

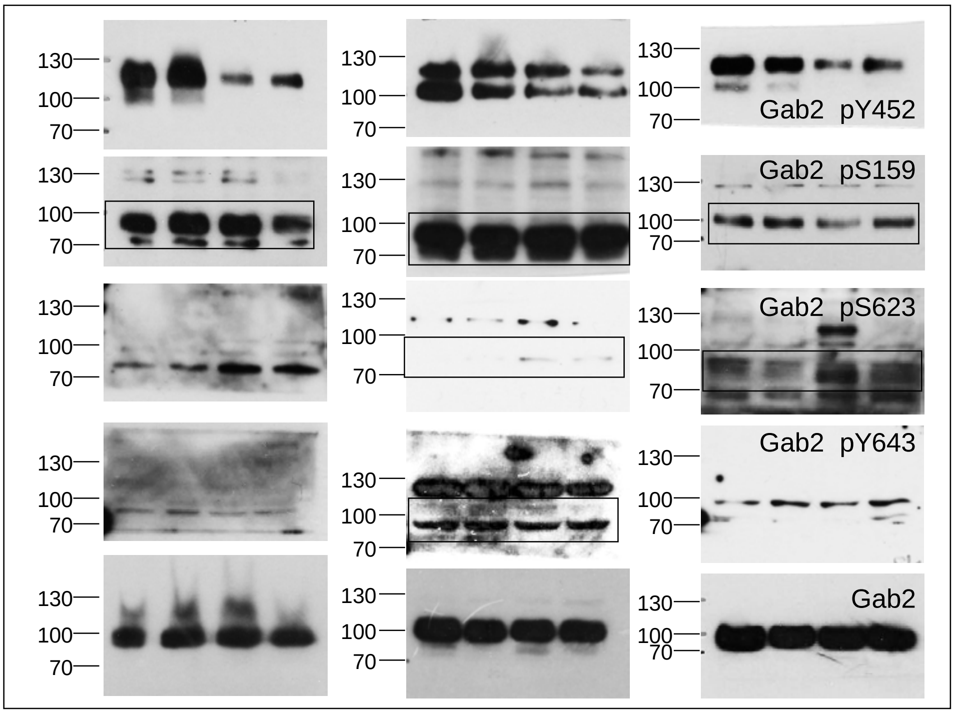

Figure 4c

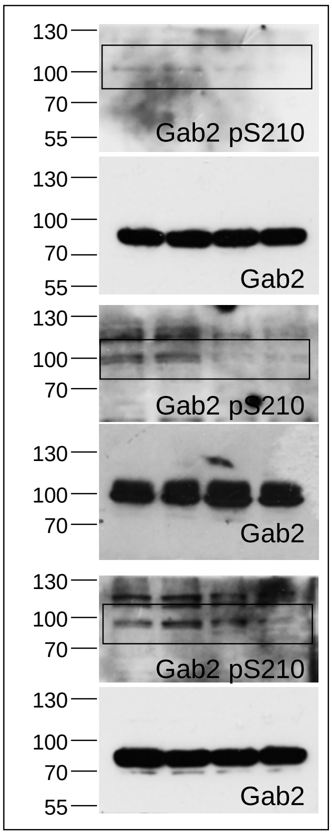

Figure 4d

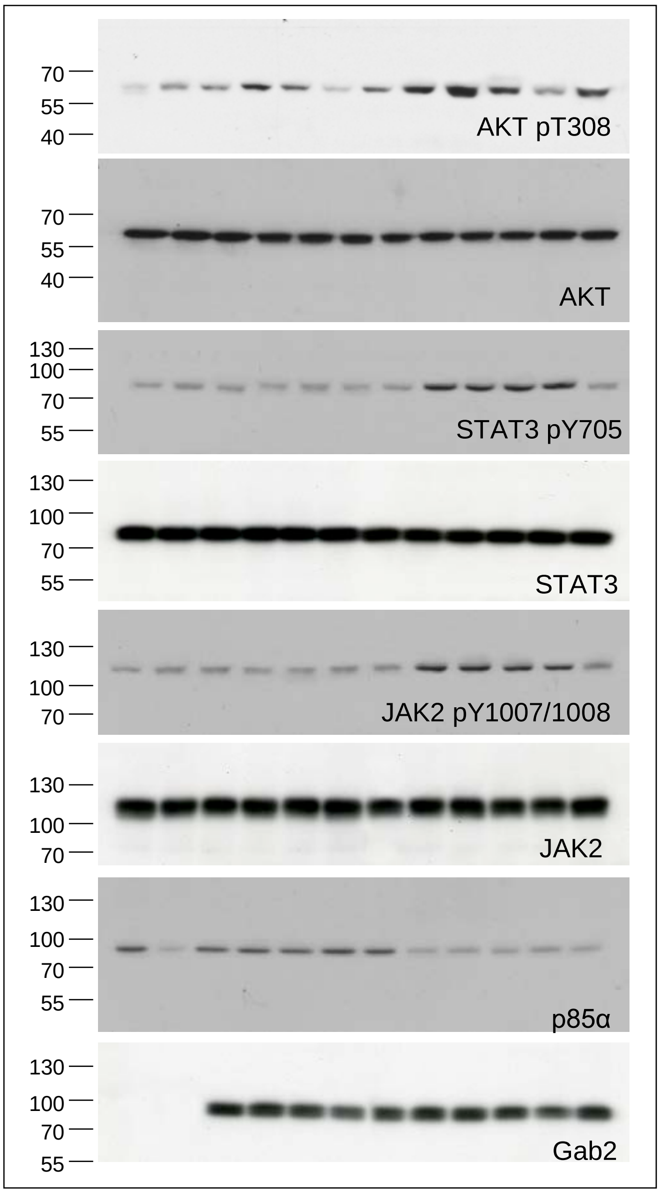

Figure 4e

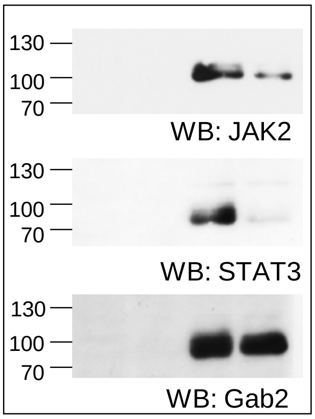

Supplementary Fig. 13. Uncropped western blots of Fig. 4.

Figure 5b

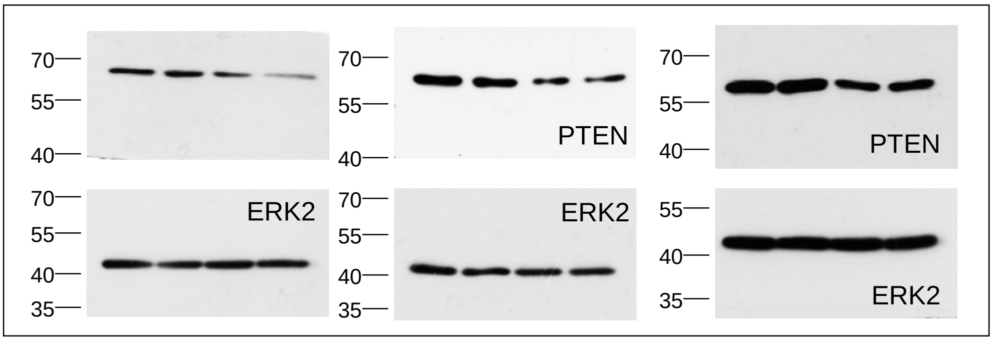

Figure 5c

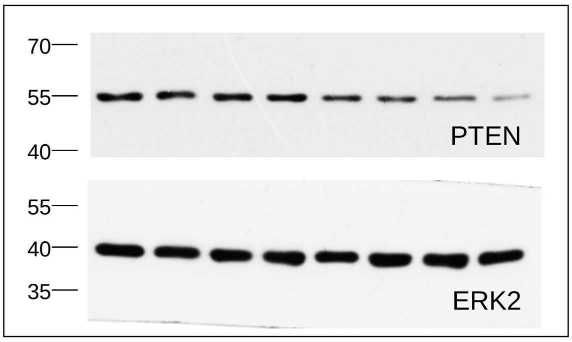

Figure 5e

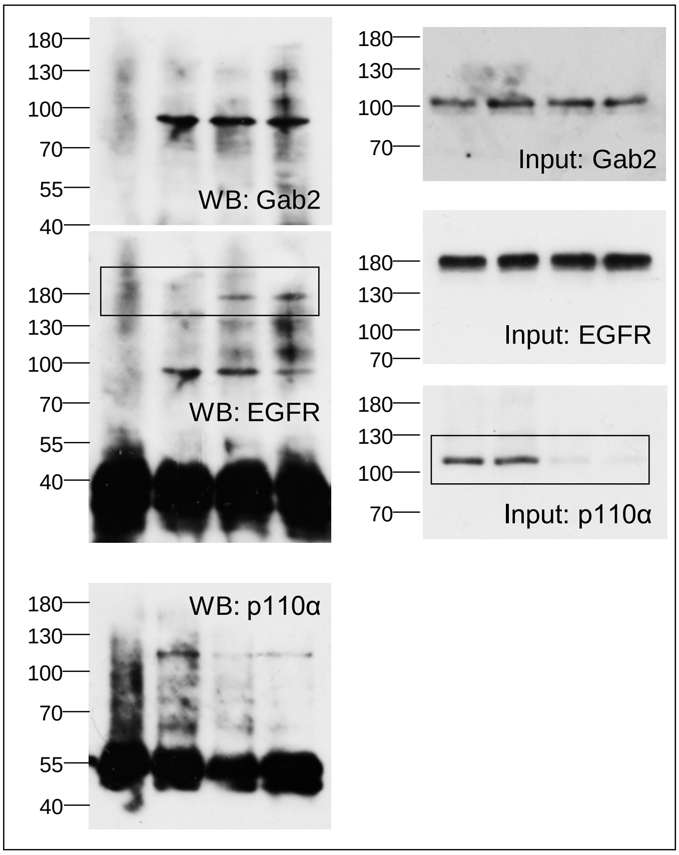

Supplementary Fig. 14. Uncropped western blots of Fig. 5.

Figure 6a

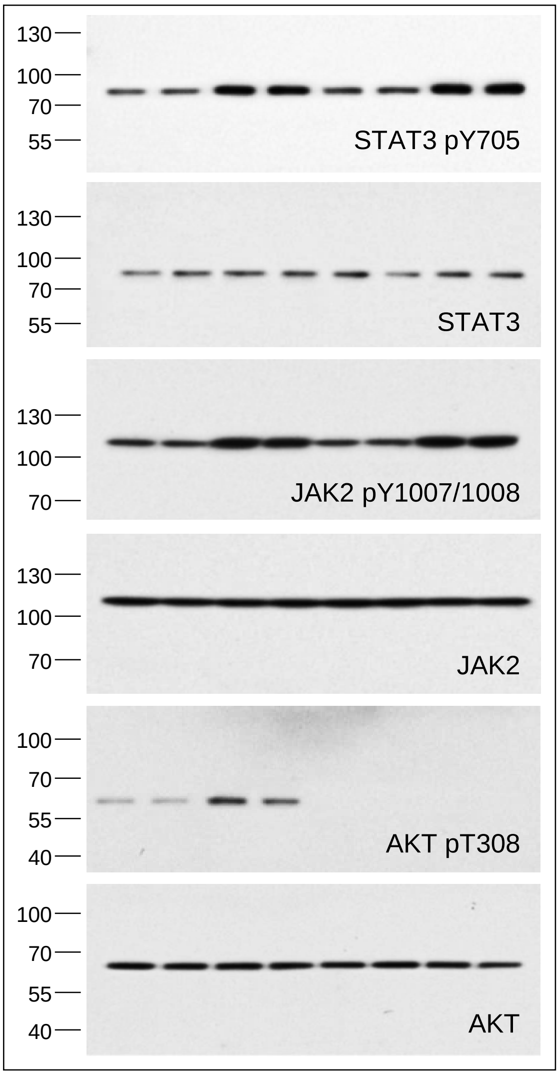

Figure 6b

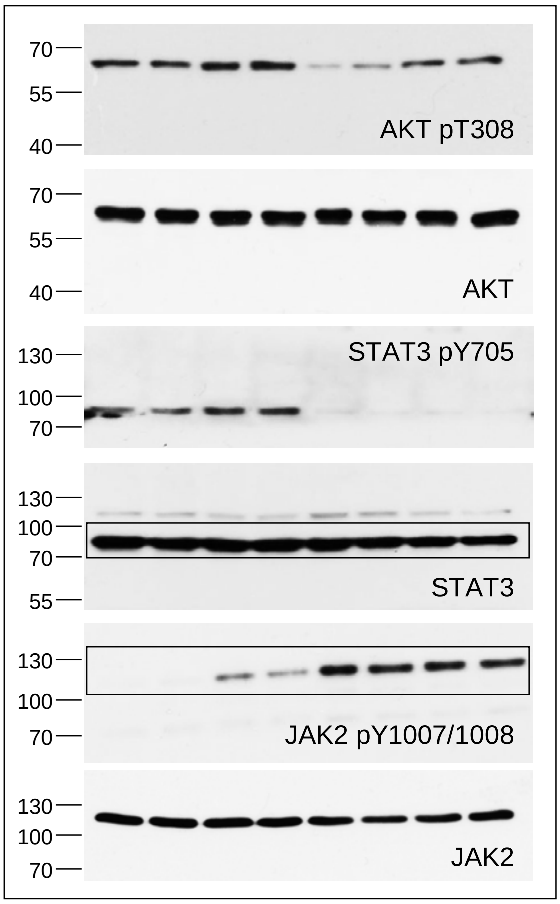

Figure 6c

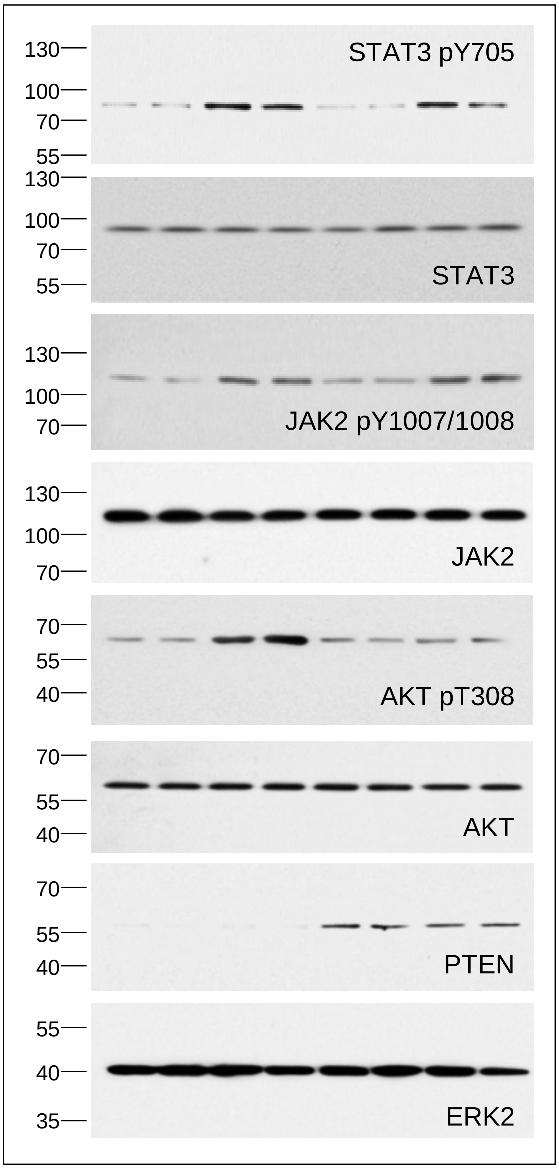

Figure 6d

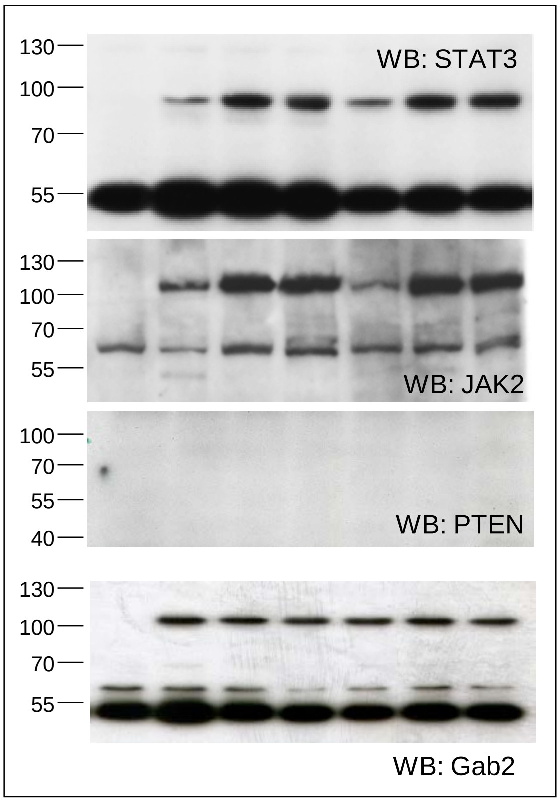

Supplementary Fig. 15. Uncropped western blots of Fig. 6.

Supplementary figure 1c

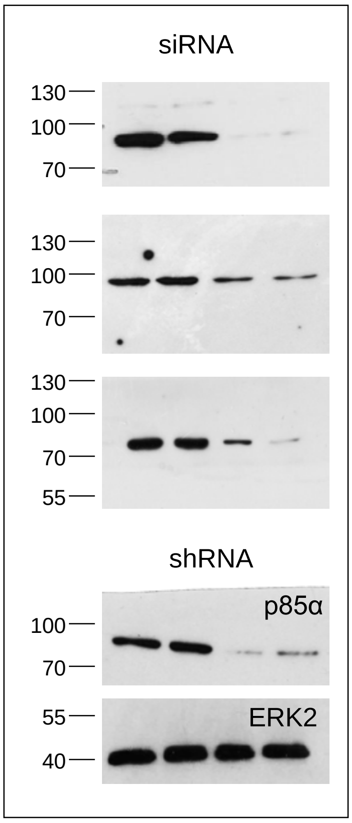

Supplementary figure 2b

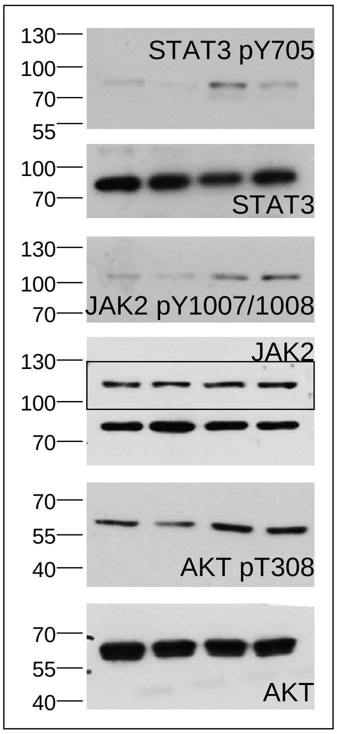

Supplementary figure 2c

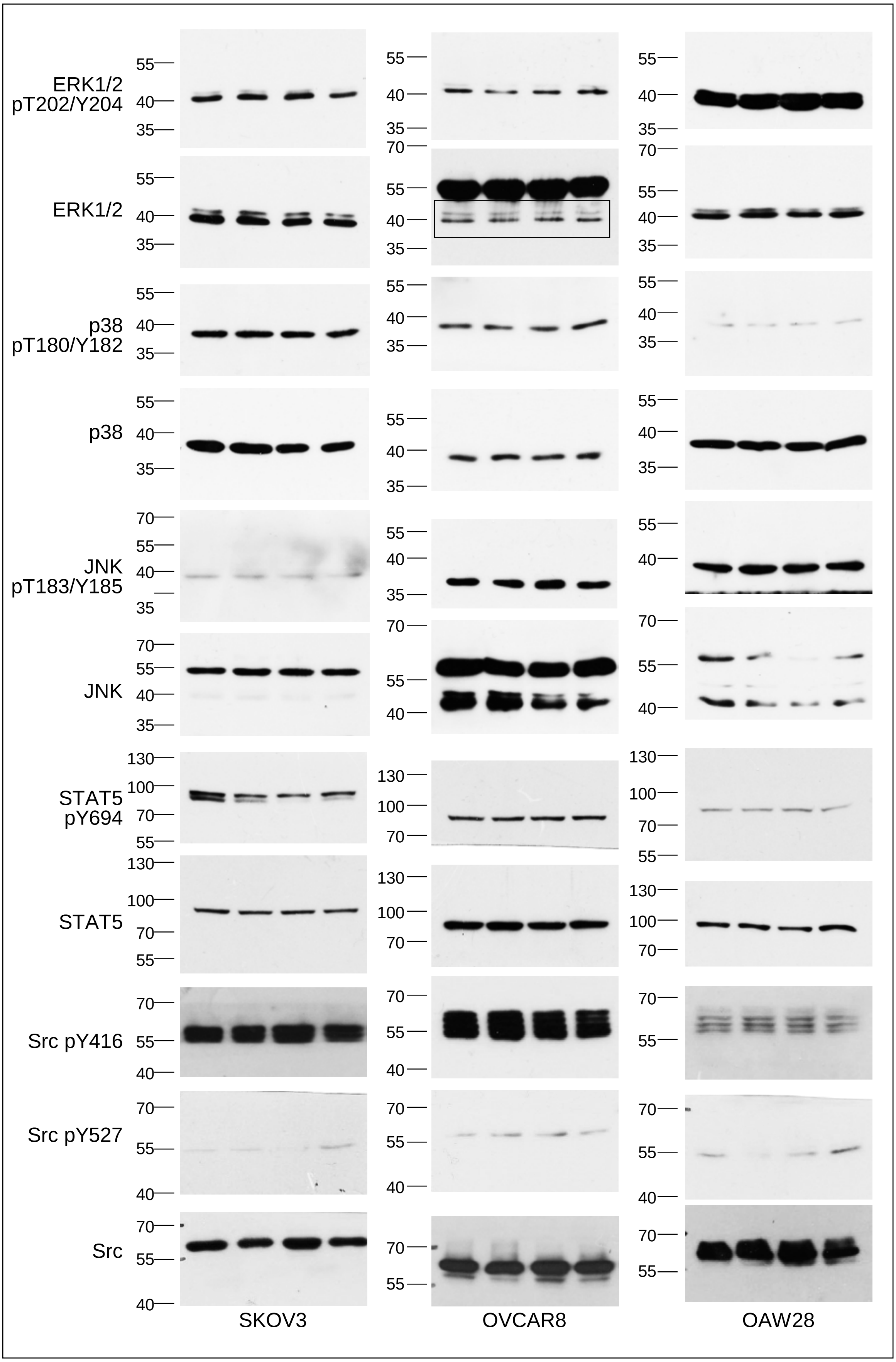

Supplementary Fig. 16. Uncropped western blots of Supplementary Fig. 1 and 2.

Supplementary figure 4a

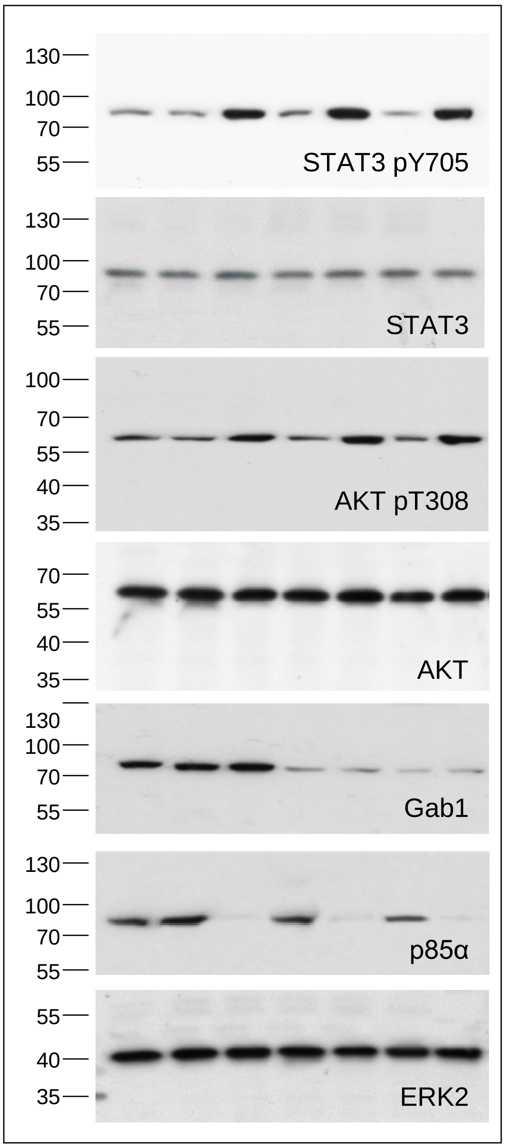

Supplementary figure 4b

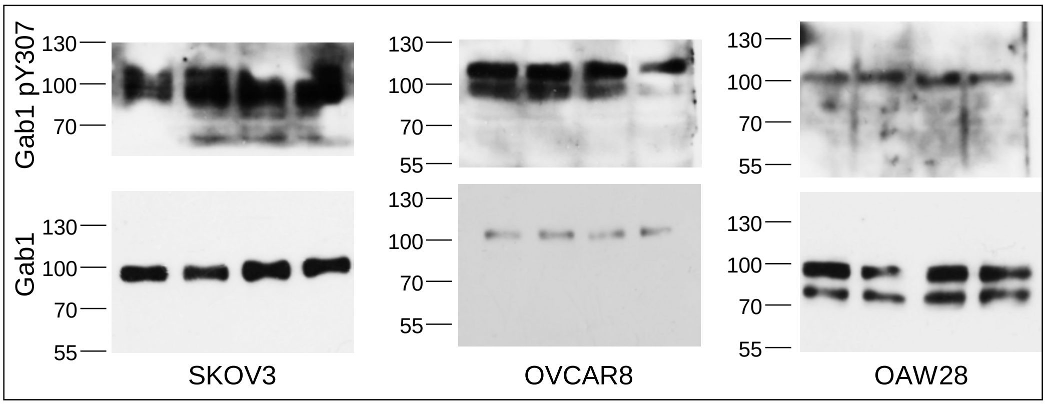

Supplementary figure 5a

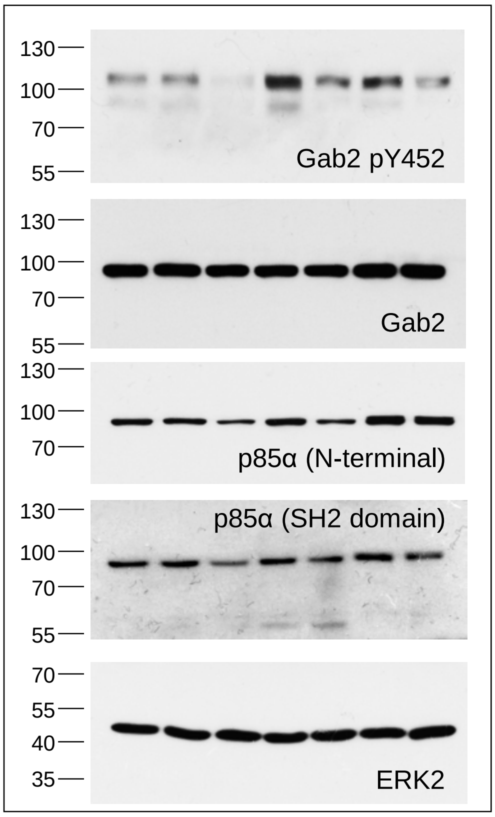

Supplementary figure 5c

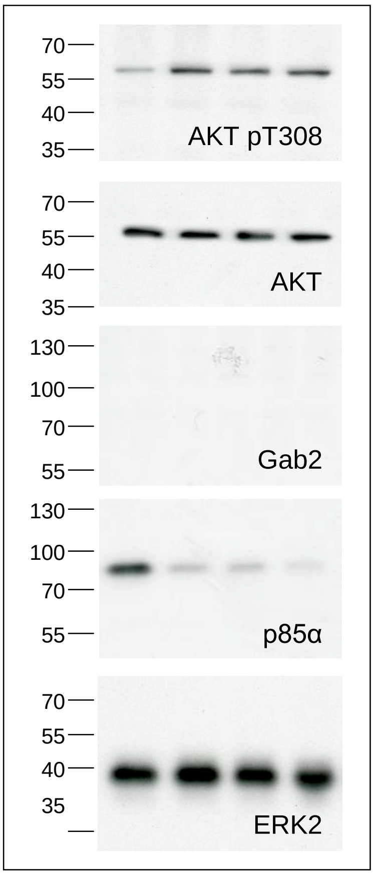

Supplementary figure 5d

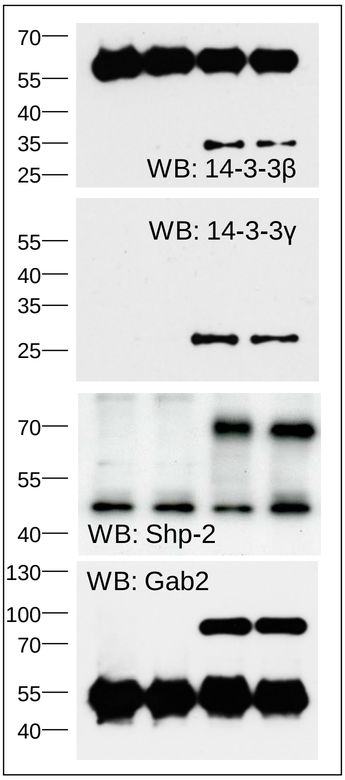

Supplementary figure 6

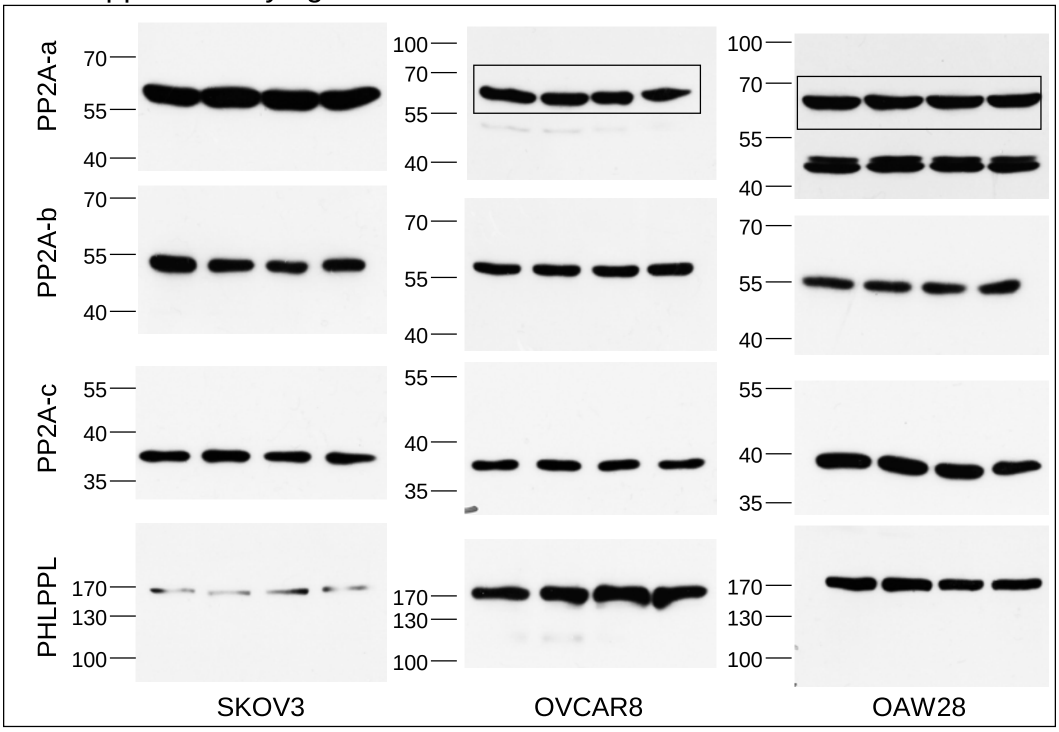

Supplementary Fig. 17. Uncropped western blots of Supplementary Fig. 4, 5 and 6.

Supplementary figure 7

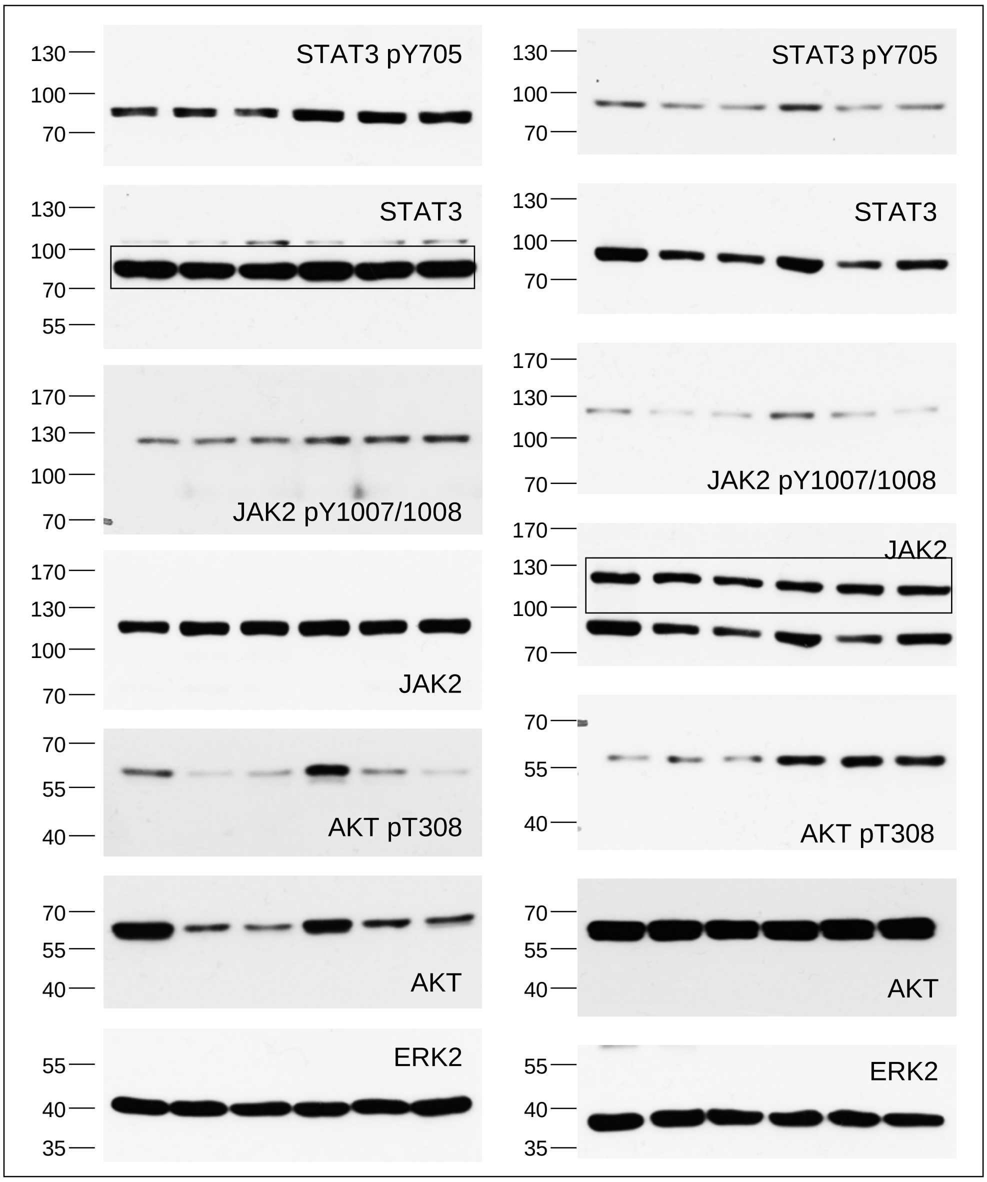

Supplementary Fig. 18. Uncropped western blots of Supplementary Fig. 7.

Supplementary figure 8a

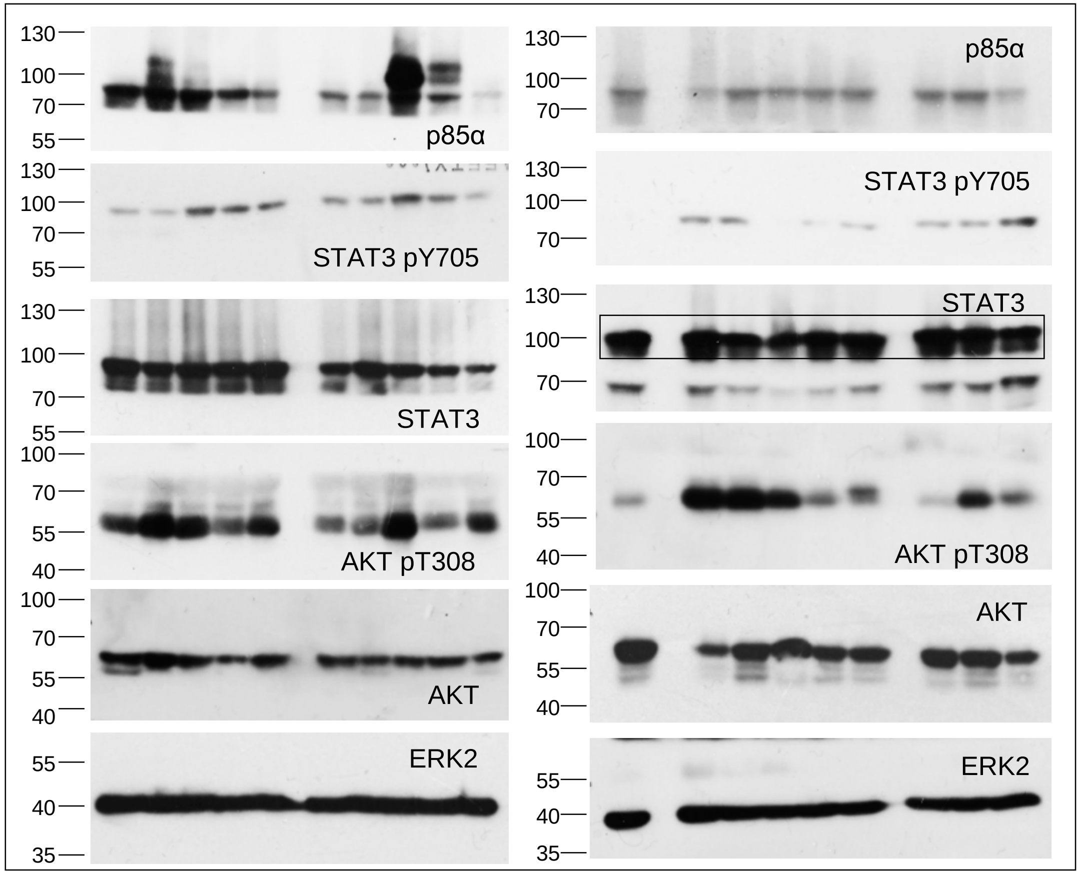

Supplementary figure 8b

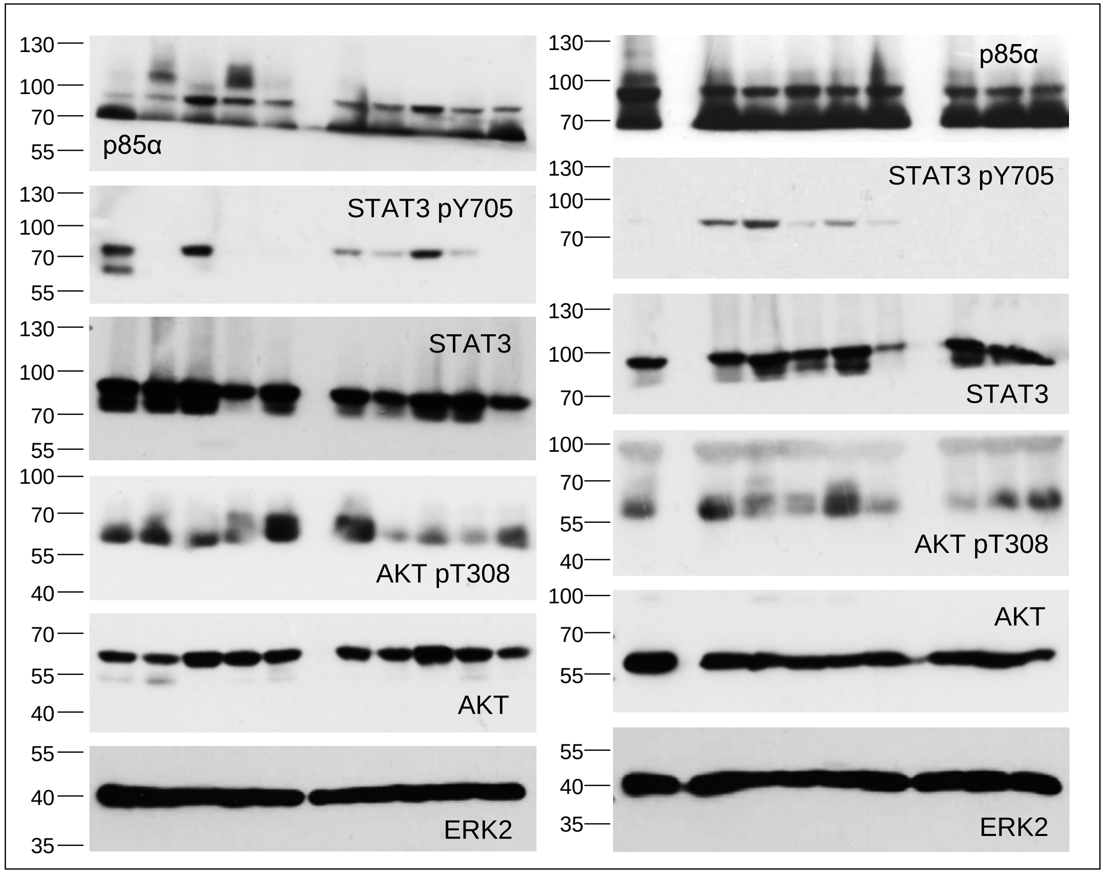

Supplementary figure 8c

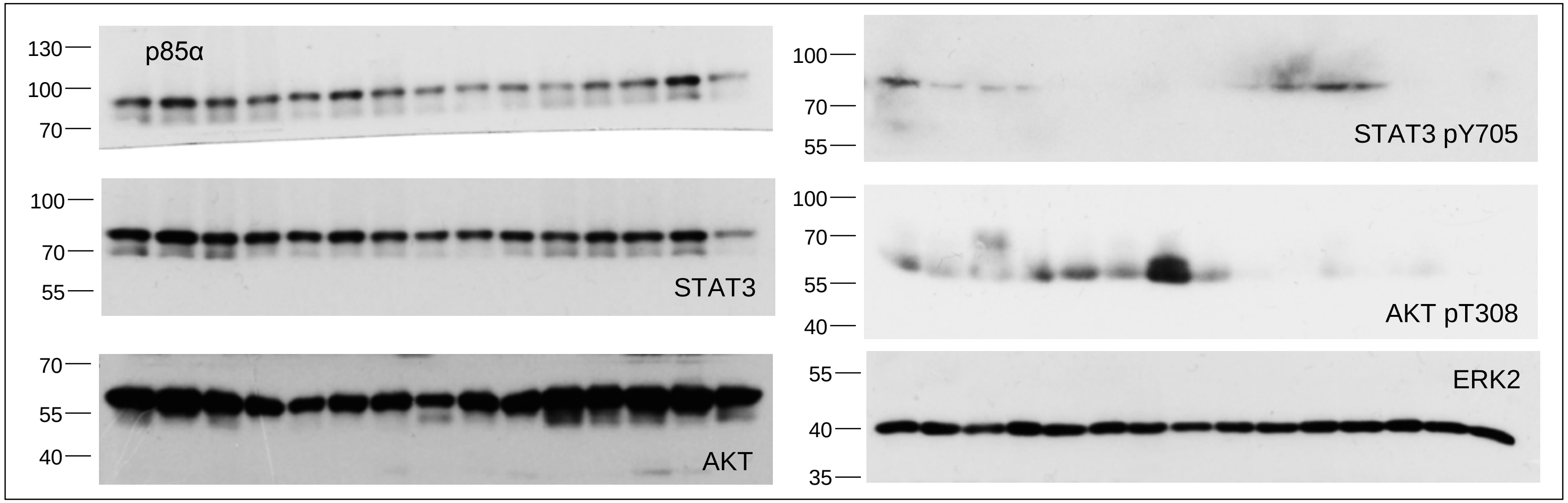

Supplementary Fig. 19. Uncropped western blots of Supplementary Fig. 9.

Supplementary Table 1. Information of siRNA and shRNA

	Gene symbol	Protein	Sequence#1	Sequence#2
siRNA	*PIK3R1* (CDS)	p85a	AGUAAAGCAUUGUGUCAUA	CCAACAACGGUAUGAAUAA
	PIK3R1 (3'UTR)	p85a	AAACUUGUCACCAUGAGAUAGCATT	
	GAB1	Gab1	GAUGCUGGAUUGACAUUUA	CAUCAAAGCUAGACACUAU
	GAB2	Gab2	UCAAGUCCAUGGCUUCUAU	GCACCGGCAGCGUUGAUUA
	STAT3	STAT3	GAGAUUGACCAGCAGUAUA	CAACAUGUCAUUUGCUGAA
	AKT1	AKT1	ACAAGGACGGGCACAUUAA	CAAGGGCACUUUCGGCAAG
	AKT2	AKT2	ACACAAGGUACUUCGAUGA	GCAAGGCACGGGCUAAAGU
	AKT3	AKT3	GCACACACUCUAACUGAAA	GAAGAGGGGAGAAUAUAUA
shRNA	*PIK3R1*	p85a	ATTCAACCACAGAACTGAAGG	ATGTCTTCTCATGATGGG

Supplementary Table 2. Information of antibodies

Name	Abbreviation	Supplier	Catalog number	WB[a] dilution	IF[b] /PLA[c] dilution	IHC[d] dilution
Anti-p85α	p85α	Santa Cruz	sc-71892	1/200		
Anti-p85α	p85α	Santa Cruz	sc-1637	1/200		1/20
Anti-p110α	p110α	Cell Signaling	#4255	1/1000		
Anti-p110β	p110β	Santa Cruz	sc-376412	1/1000		
Anti-PTEN	PTEN	Cell Signaling	#9559	1/2000		
Anti-phospho-AKT (Thr308)	AKT pS308	Santa Cruz	sc-271966	1/1000		1/20
Anti-AKT	AKT	Cell Signaling	#4691	1/3000		
Anti-phospho-STAT3 (Tyr705)	STAT3 pY705	Cell Signaling	#9145	1/1000		1/50
Anti-STAT3	STAT3	Cell Signaling	#4904	1/1000		
Anti-STAT3	STAT3	Abcam	ab119352		1/65	
Anti-phospho-STAT5 (Tyr694)	STAT5 pY694	Cell Signaling	#4322	1/1000		
Anti-STAT5	STAT5	Cell Signaling	#25656	1/1000		
Anti-phospho-JAK2 (Tyr1007/1008)	JAK2 pY1007/1008	Cell Signaling	#3776	1/1000		
Anti-JAK2	JAK2	Cell Signaling	#3230	1/1000	1/50	
Anti-JAK2	JAK2	Novus	NBP2-59451		1/50	
Anti-phospho-Gab2 (Tyr452)	Gab2 pY452	Cell Signaling	#3882	1/1000		
Anti-phospho-Gab2 (Ser159)	Gab2 pS159	Cell Signaling	#3884	1/1000		
Anti-phospho-Gab2 (Ser623)	Gab2 pS623	Invitrogen	PA5-37777	1/1000		
Anti-phospho-Gab2 (Tyr643)	Gab2 pY643	Abcam	ab78262	1/1000		
Anti-phospho-Gab2 (Ser210)	Gab2 pS210	Symansis	3043-P1	1/1000		
Anti-Gab2	Gab2	Cell Signaling	#3239	1/1000		
Anti-Gab2	Gab2	Abcam	ab32365		1/45	
Anti-phospho-Gab1 (Tyr307)	Gab1 pY307	Cell Signaling	#3234	1/1000		
Anti-Gab1	Gab1	Cell Signaling	#3232	1/1000		

Ani-ERK2	ERK2	Santa Cruz	sc-154	1/5000		
Anti-tubulin	tubulin	Cell Signaling	#2148	1/2000		
Anti-histone H3	histone H3	Cell Signaling	#9715	1/2000		
Anti-phospho-Erk1/2 MAPK (Thr202/Tyr204)	ERK1/2 pT202/Y204	Cell Signaling	#9101	1/2500		
Anti-Erk1/2 MAPK	ERK1/2	Cell Signaling	#9102	1/5000		
Anti-phospho-p38 MAPK (Thr180/Tyr182)	p38 pT180/Y182	Cell Signaling	#9211	1/1000		
Anti-p38 MAPK	p38	Cell Signaling	#9212	1/1000		
Anti-phospho-SAPK/JNK (Thr183/Tyr185)	JNK pT183/Y185	Cell Signaling	#9251	1/1000		
Anti-SAPK/JNK	JNK	Cell Signaling	#9252	1/1000		
Anti-PP2A A Subunit	PP2A-a	Cell Signaling	#2041	1/1000		
Anti-PP2A B Subunit	PP2A-b	Cell Signaling	#2290	1/1000		
Anti-PP2A C Subunit	PP2A-c	Cell Signaling	#2259	1/1000		
Anti-PHLPPL	PHLPPL	Bethyl	A300-661A-T	1/1000		
Anti-14-3-3 β	14-3-3 β	Santa Cruz	sc-25276	1/1000	1/50	
Anti-14-3-3 γ	14-3-3 γ	Santa Cruz	sc-398423	1/1000	1/45	
Anti-EGFR	EGFR	Epitomics	#1902	1/4000		
Anti-SHP-2	SHP-2	Cell Signaling	#3397	1/1000		

[a] *WB*, western blot; [b] *IF*, immunofluorescence; [c] *PLA*, proximity ligation assay; [d] *IHC*, immunohistochemistry

Supplementary Table 3. Information of primers

Gene symbol	Forward	Reverse
JUNB	GCACTAAAATGGAACAGCCCTT	GGCTCGGTTTCAGGAGTTTG
c-MYC	GCTGCTTAGACGCTGGATTT	TAACGTTGAGGGGCATCG
BCL6	CTGCAGATGGAGCATGTTGT	TCTTCACGAGGAGGCTTGAT
BCL3	CCTATACCCCATGATGTGCC	GCACCACAGCAATATGGAGA
CCNB1	AAGAGCTTTAAACTTTGGTCTGGG	CTTTGTAAGTCCTTGATTTACCATG
CCND1	TTGTGCATCTACACTGACAAC	GAAGTGTTCGATGAAATCGT
CASP4	CAAGAGAAGCAACGTATGGCA	AGGCAGATGGTCAAACTCTGTA
MCL1	CTTGCCACTTGCTTTTCTGG	CAAGGCATGCTTCGGAAACT
BCL2	GGTGGGGTCATGTGTGTGG	CGGTTCAGGTACTCAGTCATCC
MMP1	CTGGCCACAACTGCCAAATG	CTGTCCCTGAACAGCCCAGTACTTA
MMP2	GTATTTGATGGCATCGCTCA	CATTCCCTGCAAAGAACACA
MMP9	CGCTACCACCTCGAACTTTG	GCCATTCACGTCGTCCTTAT
VEGFA	GAATGCAGACCAAAGAAAGA	GACTTATACCGGGATTTCTTG
SOCS3	AGCAGCGATGGAATTACCTGGAAC	TCCAGCCCAATACCTGACACAGAA
IL6	CCAGCTATGAACTCCTTCTC	GCTTGTTCCTCACATCTCTC
PTGS2	CCCTTGGGTGTCAAAGGTAA	GCCCTCGCTTATGATCTGTC
IFNG	TCAGCTCTGCATCGTTTTGG	GTTCCATTATCCGCTACATCTGAA
GAPDH	TCCATGACAACTTTGGTATCGTG	ACAGTCTTCTGGGTGGCAGTG
