# Supplementary material for: Deregulated Gab2 phosphorylation mediates aberrant AKT and STAT3 signaling upon PIK3R1 loss in ovarian cancer
Source: Nat Commun. 2019 Feb 12;10:716. doi: 10.1038/s41467-019-08574-7 (PMC6372715; doi:10.1038/s41467-019-08574-7)
Supplement: Supplementary file 3 — Reporting Summary [file 41467_2019_8574_MOESM3_ESM.pdf]

nature research

Corresponding author(s): Lydia WT CHEUNG

Last updated by author(s): Dec 31, 2018

Reporting Summary

Nature Research wishes to improve the reproducibility of the work that we publish. This form provides structure for consistency and transparency in reporting. For further information on Nature Research policies, see Authors & Referees and the Editorial Policy Checklist.

Statistics

For all statistical analyses, confirm that the following items are present in the figure legend, table legend, main text, or Methods section.

n/a	Confirmed	
☐	☒	The exact sample size (*n*) for each experimental group/condition, given as a discrete number and unit of measurement
☐	☒	A statement on whether measurements were taken from distinct samples or whether the same sample was measured repeatedly
☐	☒	The statistical test(s) used AND whether they are one- or two-sided<br>*Only common tests should be described solely by name; describe more complex techniques in the Methods section.*
☒	☐	A description of all covariates tested
☐	☒	A description of any assumptions or corrections, such as tests of normality and adjustment for multiple comparisons
☐	☒	A full description of the statistical parameters including central tendency (e.g. means) or other basic estimates (e.g. regression coefficient) AND variation (e.g. standard deviation) or associated estimates of uncertainty (e.g. confidence intervals)
☒	☐	For null hypothesis testing, the test statistic (e.g. *F*, *t*, *r*) with confidence intervals, effect sizes, degrees of freedom and *P* value noted<br>*Give P values as exact values whenever suitable.*
☒	☐	For Bayesian analysis, information on the choice of priors and Markov chain Monte Carlo settings
☒	☐	For hierarchical and complex designs, identification of the appropriate level for tests and full reporting of outcomes
☒	☐	Estimates of effect sizes (e.g. Cohen's *d*, Pearson's *r*), indicating how they were calculated

Our web collection on statistics for biologists contains articles on many of the points above.

Software and code

Policy information about availability of computer code

Data collection	No software code was used for data collection.
Data analysis	Statistical analyses were performed with GraphPad Prism 7. Commercially available softwares, including Maxquant version 1.6.0.1 and Perseus version 1.6.0.2, were used to analyze raw mass spectrometry data.

For manuscripts utilizing custom algorithms or software that are central to the research but not yet described in published literature, software must be made available to editors/reviewers. We strongly encourage code deposition in a community repository (e.g. GitHub). See the Nature Research guidelines for submitting code & software for further information.

Data

Policy information about availability of data

All manuscripts must include a data availability statement. This statement should provide the following information, where applicable:

- Accession codes, unique identifiers, or web links for publicly available datasets
- A list of figures that have associated raw data
- A description of any restrictions on data availability

All relevant data are available from the corresponding author upon reasonable request.

Field-specific reporting

Please select the one below that is the best fit for your research. If you are not sure, read the appropriate sections before making your selection.

☒ Life sciences ☐ Behavioural & social sciences ☐ Ecological, evolutionary & environmental sciences

Life sciences study design

All studies must disclose on these points even when the disclosure is negative.

Sample size	No statistical method was used to predetermine sample size. Sample size was chosen based on the time, cost and resources available for this study.
Data exclusions	No data were excluded from the analysis.
Replication	All attempts to replicate the experimental findings were successful.
Randomization	The allocation of cells/mice to different treatments was completely random.
Blinding	Immunohistochemical staining analysis of patient tissues was blinded.

Reporting for specific materials, systems and methods

We require information from authors about some types of materials, experimental systems and methods used in many studies. Here, indicate whether each material, system or method listed is relevant to your study. If you are not sure if a list item applies to your research, read the appropriate section before selecting a response.

Materials & experimental systems

n/a	Involved in the study	
☐	☒	Antibodies
☐	☒	Eukaryotic cell lines
☒	☐	Palaeontology
☐	☒	Animals and other organisms
☐	☒	Human research participants
☒	☐	Clinical data

Methods

n/a	Involved in the study	
☒	☐	ChIP-seq
☐	☒	Flow cytometry
☒	☐	MRI-based neuroimaging

Antibodies

Antibodies used	The antibodies used in this study are listed in Supplementary Table 2.
Validation	All antibodies that are commercially available have been tested for species reactivity and application by the manufacturers.

Eukaryotic cell lines

Policy information about cell lines

Cell line source(s)	SKOV3 American Type Culture Collection; OVCAR8 and OVCAR5 National Cancer Institute; OAW28 European Collection of Authenticated Cell Cultures.
Authentication	Cell line authentication was performed by short tandem repeat (STR)-profiling.
Mycoplasma contamination	All cell lines were tested negative for mycoplasma contamination.
Commonly misidentified lines (See ICLAC register)	No misidentified cell lines were used in this study.

Animals and other organisms

Policy information about studies involving animals; ARRIVE guidelines recommended for reporting animal research

Laboratory animals	Female nude mice of 6-week old were from Charles River Lab, USA.
Wild animals	N.A.
Field-collected samples	This study did not involve samples collected from the field.
Ethics oversight	Committee on the Use of Live Animals in Teaching and Research of University of Hong Kong.

Note that full information on the approval of the study protocol must also be provided in the manuscript.

Human research participants

Policy information about studies involving human research participants

Population characteristics	All patients analyzed in this study were diagnosed with serous ovarian cancer between 1990 and 2005 in Queen Mary Hospital Hong Kong. There were no significant differences in age and clinical cancer stage.
Recruitment	The tumor samples were primary tumors collected for diagnosis before treatment. These archival tissue blocks were with confirmed diagnosis of serous ovarian cancer. The inclusion of tumor tissues was random.
Ethics oversight	Institutional Review Board of the University of Hong Kong/Hospital Authority Hong Kong West Cluster (HKU/HA HKW IRB).

Note that full information on the approval of the study protocol must also be provided in the manuscript.

Flow Cytometry

Plots

Confirm that:

☒ The axis labels state the marker and fluorochrome used (e.g. CD4-FITC).

☒ The axis scales are clearly visible. Include numbers along axes only for bottom left plot of group (a 'group' is an analysis of identical markers).

☒ All plots are contour plots with outliers or pseudocolor plots.

☒ A numerical value for number of cells or percentage (with statistics) is provided.

Methodology

Sample preparation	Please see methods "Cell cycle assay" and "Apoptosis assay".
Instrument	Canto II analyzer (BD Biosciences, San Jose, CA).
Software	FlowJo software (Tree Star Inc, Ashland, OR).
Cell population abundance	10,000 cells
Gating strategy	Gating strategy is presented in Supplementary Fig. 10.

☒ Tick this box to confirm that a figure exemplifying the gating strategy is provided in the Supplementary Information.
